# Short-Term Exposure to Foodborne Xenoestrogens Affects Breast Cancer Cell Morphology and Motility Relevant for Metastatic Behavior *In Vitro*

**DOI:** 10.1021/acs.chemrestox.4c00061

**Published:** 2024-09-12

**Authors:** Giorgia Del Favero, Janice Bergen, Lena Palm, Christian Fellinger, Maria Matlaeva, András Szabadi, Ana Sofia Fernandes, Nuno Saraiva, Christian Schröder, Doris Marko

**Affiliations:** † Department of Food Chemistry and Toxicology, Faculty of Chemistry, University of Vienna, Vienna 1090, Austria; ‡ Core Facility Multimodal Imaging, Faculty of Chemistry, University of Vienna, Vienna 1090, Austria; § Vienna Doctoral School in Chemistry (DoSChem), University of Vienna, Währinger Str. 42, Vienna 1090, Austria; ∥ Computational Biological Chemistry Department, Faculty of Chemistry, University of Vienna, Vienna 1090, Austria; ⊥ Department of Pharmaceutical Sciences, Faculty of Life Sciences, University of Vienna, Vienna 1090, Austria; # Christian Doppler Laboratory for Molecular Informatics in the Biosciences, Department for Pharmaceutical Sciences, University of Vienna, Vienna 1090, Austria; ∇ CBIOS, Universidade Lusófona’s Research Center for Biosciences & Health Technologies, Lisboa 1749-024, Portugal

## Abstract

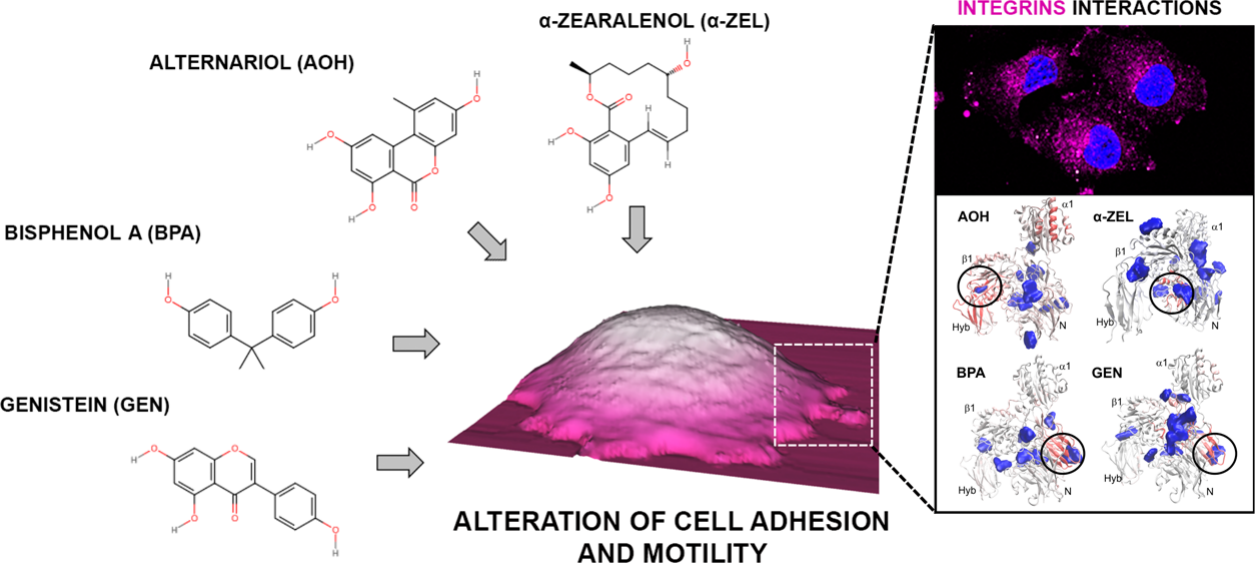

Breast cancer is highly susceptible to metastasis formation. During the time of disease progression, tumor pathophysiology can be impacted by endogenous factors, like hormonal status, as well as by environmental exposures, such as those related to diet and lifestyle. New lines of evidence point toward a potential role for foodborne endocrine disruptive chemicals in this respect; however, mechanistic understanding remains limited. At the molecular level, crucial steps toward metastasis formation include cell structural changes, alteration of adhesion, and reorganization of cytoskeletal proteins involved in motility. Hence, this study investigates the potential of dietary xenoestrogens to impact selected aspects of breast cancer cell mechanotransduction. Taking the onset of the metastatic cascade as a model, experiments focused on cell-matrix adhesion, single-cell migration, and adaptation of cell morphology. Dietary mycoestrogens alternariol (AOH, 1 μM) and α-zearalenol (α-ZEL, 10 nM), soy isoflavone genistein (GEN, 1 μM), and food packaging plasticizer bisphenol A (BPA, 10 nM) were applied as single compounds or in mixtures. Pursuing the hypothesis that endocrine active molecules could affect cell functions beyond the estrogen receptor-dependent cascade, experiments were performed comparing the MCF-7 cell line to the triple negative breast cancer cells MDA MB-231. Indeed, the four compounds functionally affected the motility and the adhesion of both cell types. These responses were coherent with rearrangements of the actin cytoskeleton and with the modulation of the expression of integrin β1 and cathepsin D. Mechanistically, molecular dynamics simulations confirmed a potential interaction with fragments of the α1 and β1 integrin subunits. In sum, dietary xenoestrogens proved effective in modifying the motility and adhesion of breast cancer cells, as predictive end points for metastatic behavior *in vitro*. These effects were measurable after short incubation times (1 or 8 h) and contribute to shed novel light on the activity of compounds with hormonal mimicry potential in breast cancer progression.

## 1 Introduction

Breast cancer belongs to the most often diagnosed cancers globally and is the most frequently diagnosed cancer in women.^1,2^ In addition to research on pharmacological treatments, the need for a better comprehension of risk factors, including the lifestyle and diet, is a pressing priority. Particularly, this opens the question of the potential role of food constituents and environmental contaminants in shaping cancer risk and aggressiveness. Among the molecules that can potentially play a role in this context, endocrine disruptive chemicals (EDCs) are raising more and more concern.^3,4^ EDCs are defined by their impact on the physiological balance of the endocrine system. These molecules can affect the hormonal axes by acting as xenoestrogens, thus mimicking endogenous estrogenic activity or antagonizing the respective signaling pathways.^5,6^ Potential exposure to EDCs is extensive, including drinking water, food packaging, nutritional supplements, or food contaminants.^7−9^ In addition to the interaction with hormonal receptors, other mechanisms of action are emerging for this class of compounds. Indeed, even if increased exposure to EDCs was already correlated to breast cancer risk and metastasis,^10^ this could be only partially traced back to alterations in estrogen receptor (ER) signaling. For example, bisphenol A (BPA) can trigger intracellular signaling with activation of ERK1/2, independent from the expression of ERs in breast cancer cells.^11^ To add complexity to the evaluation of xenoestrogens, on the one hand, industrial chemicals are described to be detrimental to the reproductive system,^12,13^ yet in contrast to this, food-derived structural analogues to female sex hormones are appreciated for their health-promoting effects.^14^ Especially considering phytoestrogens, studies correlating use/exposure to breast cancer progression depict a complex picture.^15−18^

**Figure 1:**
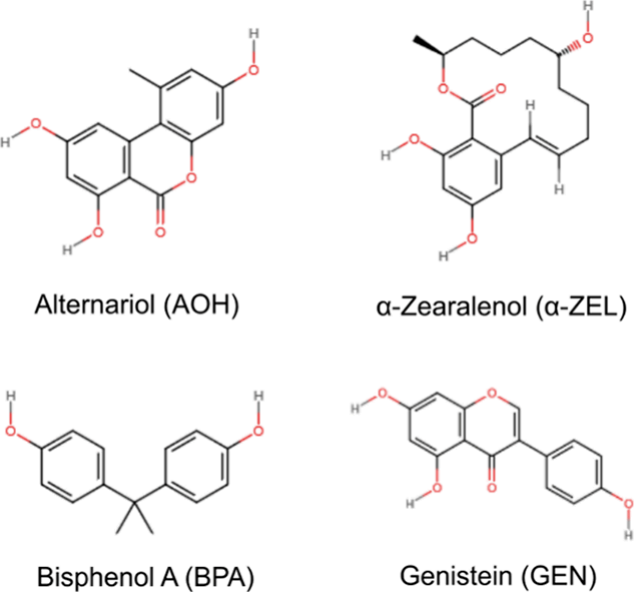
Chemical structures of selected xenoestrogens alternariol (AOH), genistein (GEN), bisphenol A (BPA), and α-zearalenol (α-ZEL).

On these molecular premises, the research focus of this study was posed on four molecules endorsing both ubiquitous environmental and dietary-derived exposures and endocrine disrupting potential (Figure 1).

Activity profiling was tailored on a panel of assays meant to reproduce crucial steps *in vitro* for the formation of a metastatic clone. The plasticizer bisphenol A (BPA) was selected for its wide use in food packaging materials^19^ and because it was previously reported to act on the estrogen receptor ligand binding domain in estrogen-sensitive MCF-7 breast cancer cells.^20^ Additionally, in MDA MB-231, BPA was suggested to affect migration via a G protein-coupled estrogen receptor.^21^ The phytoestrogen genistein (GEN) is a major component of soy-based food supplements, which was found in plasma at concentrations up to 2831 nmol/L and reported to partly accumulate in reproductive organs after intake.^22^ In addition to BPA and GEN, mycotoxins contaminating food commodities also account for xenoestrogens of increasing toxicological relevance; among these, zearalenone (ZEN), its metabolite α-zearalenol (α-ZEL), and alternariol (AOH) were already described for their xenoestrogenic potential.^23−26^ Importantly, previous studies demonstrated the systemic distribution of these molecules, underpinning potential exposure of breast tissue. Along these lines, a recent biomonitoring study revealed the occurrence of AOH, α-ZEL, and GEN (mean concentrations 0.61, 0.07, and 168 ng/mL, respectively) in the urine of primary school children, further supporting the ubiquitous presence of these molecules.^27^ Additionally, Braun and colleagues described the occurrence of ZEN in Nigerian as well as Austrian pooled samples of breast milk; this clearly indicates that ZEN can reach the breast tissue, possibly already exposing even infants through breast milk,^28,29^ making it plausible for its metabolite α-ZEL to come into contact with breast cells. Similarly, BPA has been detected in breast milk in several studies worldwide, i.e., with a mean concentration 0.444 μg/L reported in China^30^ or median concentration 1.03–0.69 μg/L in South Africa.^31^

Considering the high likelihood of coexposure to endocrine disruptors on a daily basis, the activity of the compounds as single molecules and in selected mixtures was included. As a working hypothesis, the possibility that xenoestrogens could mimic hormonal-mediated functions beyond the binding to estrogen receptors was explored. For instance, 17-β-estradiol (E2) participates in integrin tether during blastocyst intrauterine binding, making cell adhesion dependent on the hormonal status.^32^ Considering the structural analogies between E2 and the endocrine disruptors, it can be postulated that the adhesion molecules and the cytoskeletal network could become molecular targets for xenoestrogens.^33^ Building on this, this study aimed to investigate the potential of dietary and environmental xenoestrogens to perturb cell adhesion and motility. Both aspects are essential for cancer aggressiveness and metastasis formation including detachment from the primary tumor mass, migration, and adhesion to the blood or lymphatic vessels necessary for extravasation. Postulating molecular pathways independent from the estrogen receptors activations, we selected an estrogen sensitive cell line (MCF-7) and a triple negative breast cancer (TNBC, MDA MB-231), which does not express the estrogen receptor, the progesterone receptor or the epidermal growth factor receptor 2.^34^ Along this line, we privileged short-term exposures (from 1 to 8 h), which are less dependent on gene transcription and signaling cascades thereof and better mirror discontinuous and/or limited bioavailability of the chemicals after dietary or lifestyle related uptake.

## 2 Materials and Methods

### 2.1 Chemicals and Other Materials

Cell culture plasticware was purchased at Sarstedt AG & Co (Nuembrecht, Germany) and microscopy plastics and glassware at ibidi (Graefeling, Germany). Cell growth media and supplements were bought from Gibco Thermo Fisher Scientific (Waltham, MA, USA), Sigma-Aldrich Chemie GmbH & Co (Steinheim, Germany), and Szabo Scandic (Vienna, Austria). CellTiter-Blue (10×) concentrate was obtained from Promega (Waldorf, Germany). Sulforhodamine B (SRB), AOH, α-ZEL, BPA, GEN, Fulvestrant (ICI 182.780, ICI), and nocodazole (noc) were from Sigma-Aldrich Chemie GmbH & Co (Steinheim, Germany). Dimethyl sulfoxide (DMSO), Triton X-100, trichloroacetic acid (TCA), paraformaldehyde solution (37%), and ROTI-Mount FluorCare DAPI were purchased at Carl Roth (Karlsruhe, Germany). MatrigelMatrix was bought from Corning (Glendale, AZ, USA). Immunofluorescence antibodies were bought at abcam (Cambridge, UK): anti-activated integrin β1 (ab30394, mouse), anti-cathepsin D (ab75852, rabbit), and phalloidin molecular probe (Thermo Fisher Scientific: Oregen Green 488, 07466).

### 2.2 Cell Lines and Experimental Setup

MCF-7 breast cancer cells and MDA MB-231 (both ATCC, Manassas, VA, USA) invasive phenotype breast cancer cells were cultivated in DMEM high glucose supplemented with 10% fetal bovine serum and 1% penicillin/streptomycin (Pen/Strep). In addition, for growth conditions of MCF-7, 0.1 μg/mL insulin solution (bovine) was added to the culture medium. Cells were maintained at 37 °C, 5% CO_2_, and 95% humidity, passaged twice a week, and regularly checked for mycoplasma contamination. As previously described for the study of molecules with potential endocrine disrupting activity,^24,26,35−37^ the culture medium (CM) was exchanged for the experimental medium (EM) composed of DMEM (phenol red free) supplemented with 10% dextran-treated charcoal-stripped fetal calf serum (CD-FCS) (Thermo Fisher Scientific, Waltham, USA) and 100 U/mL Pen/Strep for MCF-7 and MDA MB-231. Stock solutions of compounds for incubations were prepared in DMSO (Carl Roth); accordingly, a corresponding solvent control was included in all experiments. Endocrine active chemicals of dietary sources chosen for this study include the *Alternaria alternata* mycotoxin AOH (1 μM), the *Fusarium spp* human mycotoxin metabolite α-ZEL (10 nM), the packaging compound BPA (10 nM), and the isoflavone GEN (1 μM). Concentrations were chosen to maintain high cell viability MCF-7 and MDA MB-231 (above 80%; Supplementary Figure 1) and based on previous studies describing the activity profiling of the estrogenic activity.^26,35,38^ Control substances for mechanistic studies included the compound nocodazole, disruptive of microtubule assembly, the female sex hormone and agonist of estrogen receptors (ER) 17-β-estradiol (E2; 1 nM), and the full ER antagonist ICI (1 μM). Cell monolayers were grown as indicated in the respective sections, and incubations with the EM was dependent on the experimental layout.

### 2.3 Cell Viability

Cells were seeded with the culture medium at a density of 15 000 cells/cm^2^ for MCF-7 and 18 000 cells/cm^2^ for MDA MB-231 for 24 h. Afterward, incubation solutions prepared in an experimental medium were added to the adherent cells for 8 h. Subsequently, experimental media were aspirated, and a 1:10 dilution of CellTiter-Blue (Promega) in DMEM with no phenol red was added to the cells for 4 h. Fluorescence readout of the supernatant was obtained using a Cytation5 (Biotek, Agilent Technologies, Santa Clara, CA, USA) MultiMode reader utilizing 560 nm_ex_/590 nm_em_. Experiments were performed in technical triplicates for each incubation condition, and test values were normalized to solvent controls (T/C). After the performance of the CellTiter-Blue assay, cell monolayers were immediately fixed by applying 5% TCA in dH_2_O for 30 min at 4 °C. After a washing step (PBS), cell proteins were stained using 0.4% SRB solution in dH_2_O overnight at room temperature (RT). Subsequently, SRB was washed away using dH_2_O and 1% acetic acid, and cells were dried for 24 h (RT). Afterward, protein-bound SRB was solubilized in 10 mM TRIS (pH 10) (orbital shaking at 500 rpm, 5 min), and the absorbance of the supernatant was measured at 570 nm.

### 2.4 Assessment of Single Cell Motility

Mimicking ECM, single cell motility was investigated on top of Matrigel Matrix rich in ECM proteins to observe cell responsiveness in migration to external cues as previously described.^39^ Before single cell random migration assessment, 96-well plates were functionalized with 0.3 mg/mL Matrigel Matrix solution in DMEM (phenol red free), supplemented with 50 μg/mL gentamicin solution. Matrigel Matrix solidified for 50 min at room temperature. Subsequently, 4500 cells per well of MCF-7 and 5400 cells per well of MDA MB-231 were seeded onto the culture plates within the EM in technical duplicates. Following incubation for 4 h, tracking of single cell movements was monitored with a Lionheart FX Automated Microscope (Biotek) at 37 °C, 5% CO_2_, and humidity for 8 h (for the quantitative analysis) and up to 20 h. Automatically, phase contrast images of the cells adhering to the Matrigel Matrix were taken every 25 min. The Image Analysis Software ImageJ and the Chemotaxis Tool were utilized to analyze the cell movement direction and speed. Forty single cells per well were randomly selected and tracked. Single cell random migration experiments were conducted in at least four individual experiments. Single cell speed readout is declared as “velocity [μm/min], and accumulated and Euclidean distances were analyzed for all technical replicates and used for calculating “directional persistence” for the 8 h time frames as follows:  as the parameter of directional continuity.^40^

### 2.5 Cell Deadhesion Assay

For the deadhesion experiments, cells were seeded at a density of 19 500 cells/cm^2^ (MCF-7) and at 23 000 cells/cm^2^ (MDA MB-231) in 48-well plates for 24 h. For the respective incubations, media were exchanged for EM. Deadhesion experiments were carried out as previously described^39^ with minor modifications in at least biological triplicates and 4–5 technical replicates. After 1 and 8 h of incubation, incubation solutions were removed, and cells were washed carefully using DMEM (phenol red free) (Gibco Thermo Fisher Scientific; Waltham, MA, USA). To trigger cell detachment, DMEM was exchanged for 1 mM EDTA solution in DPBS, and plates were placed at 37 °C, 5% CO_2_, and 95% humidity for 10 min. Subsequently, the EDTA solution was removed, and cells were fixed using 5% TCA solution for 1 h at 4 °C. After fixation, cells were washed with ddH_2_O, and the plates were dried overnight. On the following day, fixed cells were stained by applying 0.4% sulforhodamine B (SRB) solution overnight in the dark. After staining, cells were washed twice with ddH_2_0 and applied with 1% acetic acid twice. After drying the plates overnight again, SRB was dissolved in 10 mM TRIS base for 5 min while shaking. Absorbance measurements were obtained using a Cytation5 plate reader (Biotek) at 570 nm. Absorbance values for solvent control conditions not treated with EDTA were set to 100% of attached cells for each cell line.

### 2.6 Cell Adhesion/Spread Assay

Prior to the assay (24 h), the culture medium for both MCF-7 and MDA MB-231 was exchanged with EM and 96-well plates (Sarstedt) were coated with l-ornithin (0.005%) overnight. Thereafter, the plates were washed with sterile water and treatments (100 μL) in EM added to yield a final concentration (calculating 100 μL cell suspension to be added after calcein staining) of 0.1 μM, 1 μM, and 10 μM (AOH and GEN) or 1 nM, 10 nM, and 100 nM (α-ZEL and BPA). A 1 mL cell suspension in estrogen-free medium was prepared for each cell line containing 1.2 million cells. These were stained with 5 μL of calcein AM (1 mM) and incubated for 30 min at 37 °C, the supernatant was removed, and the cells were resuspended in 1 mL of medium and spun down to a pellet. This washing step was repeated three times. The cells were then resuspended in 5 mL of estrogen-deprived medium, 100 μL of cell suspension (corresponding to 25 000 cells) was added to each well, and the 96-well plate was incubated for 1 h at 37 °C, 5% CO_2_, to allow for cells to attach. After incubation, nonadherent cells were gently removed, the plate was washed four times with prewarmed normal external solution (NES^41^), and finally, 200 μL of NES-buffer was added to each well for live cell imaging. Thereafter, cells were imaged with a 10× magnification using the GFP channel (489 nm_ex_/508 nm_em_) implementing a Lionheart FX automated microscope (BioTek Instruments Inc., Winooski, VT, USA) and a minimum of three different optical fields were used for analysis of each condition. Experiments were performed in three independent cell preparations (biological replicates), and at least 12 optical fields were analyzed for each data set.

### 2.7 Immunofluorescence Imaging

Immunofluorescence staining and imaging were performed as previously described with minor modifications.^42^ Cells were seeded onto 8-well μ-dishes (ibidi) at a density of 2000 cells (MCF-7) and 2500 cells (MDA MB-231) per well for 24 h. For the treatments, compounds diluted in EM were applied to the cell monolayer for 8 h. Afterward, cells were washed using PBS-A and fixed by applying 3.5% FA in PBS for 10 min. Cell permeabilization was achieved by applying 0.2% Triton X-100 in PBS-A for 12 min followed by a washing step and blocking unspecific binding sites with 1% BSA solution in PBS-A for 1 h. Primary antibody dilutions in 0.25% BSA solution were incubated for 2 h (cathepsin D 1:250, integrin β1 1:250, phalloidin 1:500). Afterward, cells were washed with 0.05% Triton X-100 in PBS-A three times and once with PBS-A prior to incubation with secondary antibodies for 1.5 h (1:1000 in 0.25% BSA in PBS-A). Subsequently, cell monolayers were washed several times, stainings were post-fixed (4% FA in PBS-A), cells were washed again, and FA quenched by applying a 100 mM glycine solution. Immunofluorescence images were acquired using a LSM Zeiss 710 microscope, coupled to an ELYRA PS.1 system, AndoriXon 897 (EMCCD) camera, and a Plan Apochromat 63× objective. Randomly chosen optical fields were acquired from three to five independent cell preparations measuring cell morphometric descriptors as area and circularity,^43^ as well as integrated fluorescence signal intensities (Relative fluorescence units; R.f.u. *n* ≥ 60 cells).

### 2.8 Statistical Analysis

Data were generated in at least three independent cell preparations (biological replicates). Statistical analysis was performed according to data distribution, and normally distributed data were compared using Student’s *t*-test, otherwise, the Mann–Whitney test was used. Concentration-dependent effects were analyzed applying a one-way ANOVA test with a Fisher LSD post hoc test for multiple comparisons. Significant difference among the data sets was assigned using a cutoff value (*p*-value) of 0.05.

### 2.9 Molecular Dynamics Simulations of Integrin as Target for Endocrine Disruptive Chemicals

As the molecular dynamics (MD) simulations of the full solvated protein integrin αxβ2 (RCSB pdb-code: 3k6s(44)) are computationally too expensive, only the essential domains were selected via VMD^45^ and saved with new C- and N-termini. This includes the N-domain (residues 1:126 and 328:597) and α1 domain (residues 127:327) of chain A as well as the Hyb domain (residues 58:101 and residues 343:423) and β1 domain (residues 102:342) of chain B.^46^

Since the aqueous solution of the integrin was modeled by the standard CHARMM force field,^47^ the compounds AOH, α-ZEL, GEN, BPA, and RGD (Arg-Gly-Asp; integrin-binding peptide) were parametrized via CGenFF^48^ and the standard TIP3 water model was used. Twelve independent systems (Supplementary Table 1) contained one protein, ca. 81300 water molecules, and 10 molecules of the ligands AOH, α-ZEL, GEN, BPA, and RGD as well as 0.15 M KCl. Since the protein is negatively charged, 15 excess potassium ions were added to the mixture.

The systems were set up using the CHARMM-GUI Multicomponent Assembler^49^ in a simulation box of approximately 13.7 nm length at 303 K and 1 atm. To increase the time step to four femtoseconds, hydrogen mass repartitioning was applied.^50^ The cutoff radius for the nonbonded interactions in real space was set to 1.2 nm, and the Particle Mesh Ewald method was applied with a tolerance of 0.0005. The program package OpenMM^51^ was used to generate trajectories of 200 ns of each system in Supplementary Table 1. The first three nanoseconds were considered as the equilibration period and disregarded for the subsequent trajectory analysis by the self-written Python code using the MDAnalysis package.^52^ The root-mean-square deviation (RMSD) was computed for each domain (N, α1, Hyb, and β1) in each system separately. The root-mean-square fluctuations (RMSFs) were obtained for each amino acid in these systems. Subsequently, the corresponding RMSF values of the amino acids within a particular system were assigned to the respective domain (N, α1, Hyb, and β1) and used for the box plots.

**Table 1:**
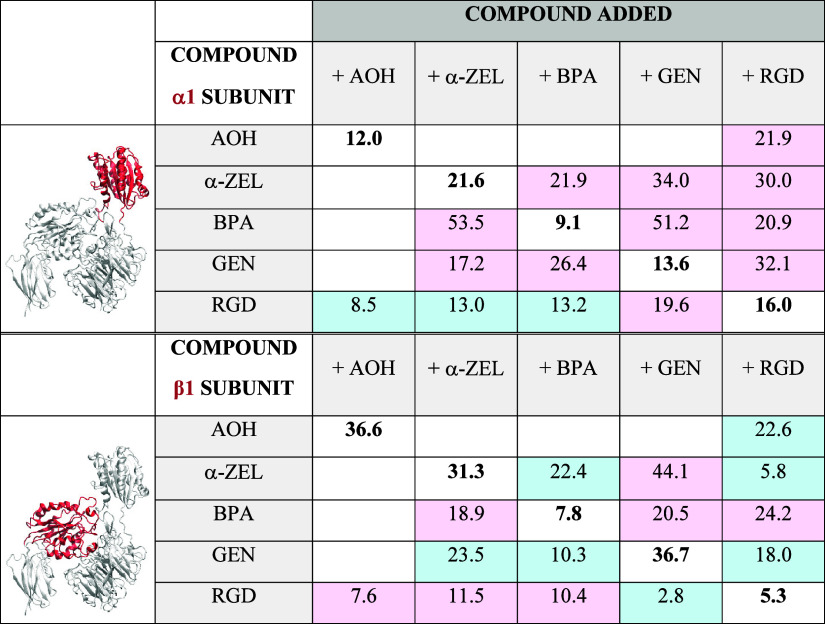
Domain Contacts *C*_domain_ of the Chemicals of Interest^a^

a Each row depicts the number of the compound fulfilling the distance criterium (0.7 nm). Each column denotes the co-compound. Bold entries in the diagonal highlight the combination of the same molecule. Light blue entries indicate the decrease in that row in comparison to diagonal values and pink entries indicate an increase.

### 2.10 Docking of Endocrine Disruptive Chemicals

The docking of the AOH, α-ZEL, GEN, BPA, and RGD was performed with SwissDock^53^ as SwissDock uses the exact same force field as our MD simulations and consequently allows for the discrimination between static binding (from docking) and dynamic binding (from our MD simulations). The binding modes are generated in the vicinity of all of the protein cavities. The results are clustered and visualized by VMD for interpretation.

## 3 Results

### 3.1 Effect of Dietary Xenoestrogens on Breast Cancer Cell Migration

**Figure 2:**
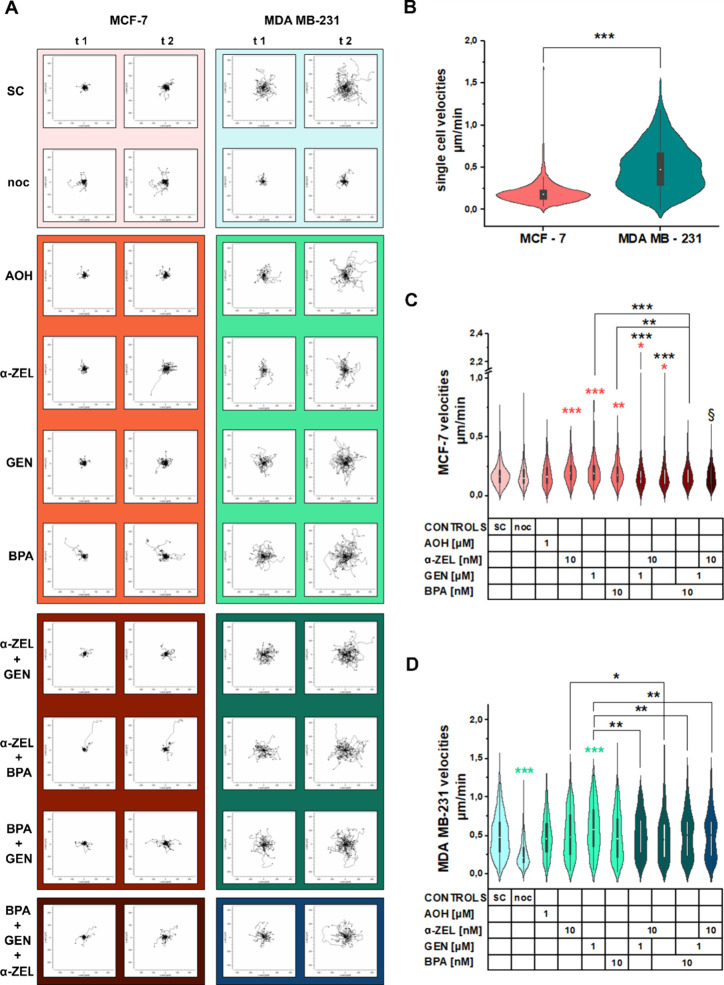
Single cell random migration. (A) Representative tracks of single cell movement over 20 h observation time (t 1 = half time and t 2 = total acquisition time). (B) Distribution plots of single cell velocities (μm/min) tracked over 8 h in control conditions (*n* ≥ 270). In the presence of selected xenoestrogens, (C) results obtained for MCF-7 cells and (D) cell motility of MDA MB-231. Significant differences to controls were calculated applying the Mann–Whitney test and indicated as *(*p* < 0.05), **(*p* < 0.01), and ***(*p* < 0.001). Black symbols are used for differences among treatments and colored symbols for differences in comparison to solvent controls. For MCF-7 (panel C), black symbols above the treatments *** α-ZEL + GEN and α-ZEL + BPA indicate significant differences to respective single treatments and § indicates that the combination α-ZEL + GEN + BPA is different from single treatments with at least *p* < 0.01. SC: solvent controls and noc: 0.1 μM nocodazole. Experiments were performed in *n* ≥ 3 independent cell preparations (biological replicates) quantifying *n* = 40 cells/biological replicate.

Considering the essential role of cell migration in metastatic spread, the impact of individual dietary xenoestrogens on random cell migration was evaluated. The two cell lines differed remarkably in the single cell speed of movement (Figure 2A,B) and directional persistence (Supplementary Figure 2), aligned to a previously published work.^54^ While the median velocity of MCF-7 was 0.2 μm/min, MDA MB-231 reached an average speed of 0.5 μm/min (Figure 2A,B). Accordingly, MDA MB-231 cells were more sensitive than MCF-7 to the application of the microtubule disruptor nocodazole (0.1 μM; Figure 2C,D), which also significantly decreased cell mass (Supplementary Figure 1D). The velocity of MCF-7 increased significantly after exposure to the single substances α-ZEL, BPA, and GEN (Figure 2A,C). No variation could be observed for cells incubated with AOH in comparison to solvent controls (Figure 2C). Selected combinations of compounds were chosen to start investigating the potential effects of mixtures in these responses. Binary combinations of α-ZEL + GEN and α-ZEL + BPA reduced cell migration velocities in comparison to controls (Figure 2C). No significant changes could be measured for cells incubated with GEN + BPA (Figure 2C). All binary treatments as well as the exposure to α-ZEL + GEN + BPA decreased cell speed compared with the incubations with single substances (Figure 2C). MDA MB-231 cells increased their speed after 8 h of exposure to GEN (1 μM). Binary and ternary combinations involving GEN could hamper this effect (Figure 2D; GEN + α-ZEL; GEN + BPA; and α-ZEL + GEN + BPA). Combinatory exposure to α-ZEL + BPA returned reduced motility in comparison to cells exposed exclusively to α-ZEL (Figure 2D).

### 3.2 Effect of Dietary Xenoestrogens on Breast Cancer Cell Adhesion

**Figure 3:**
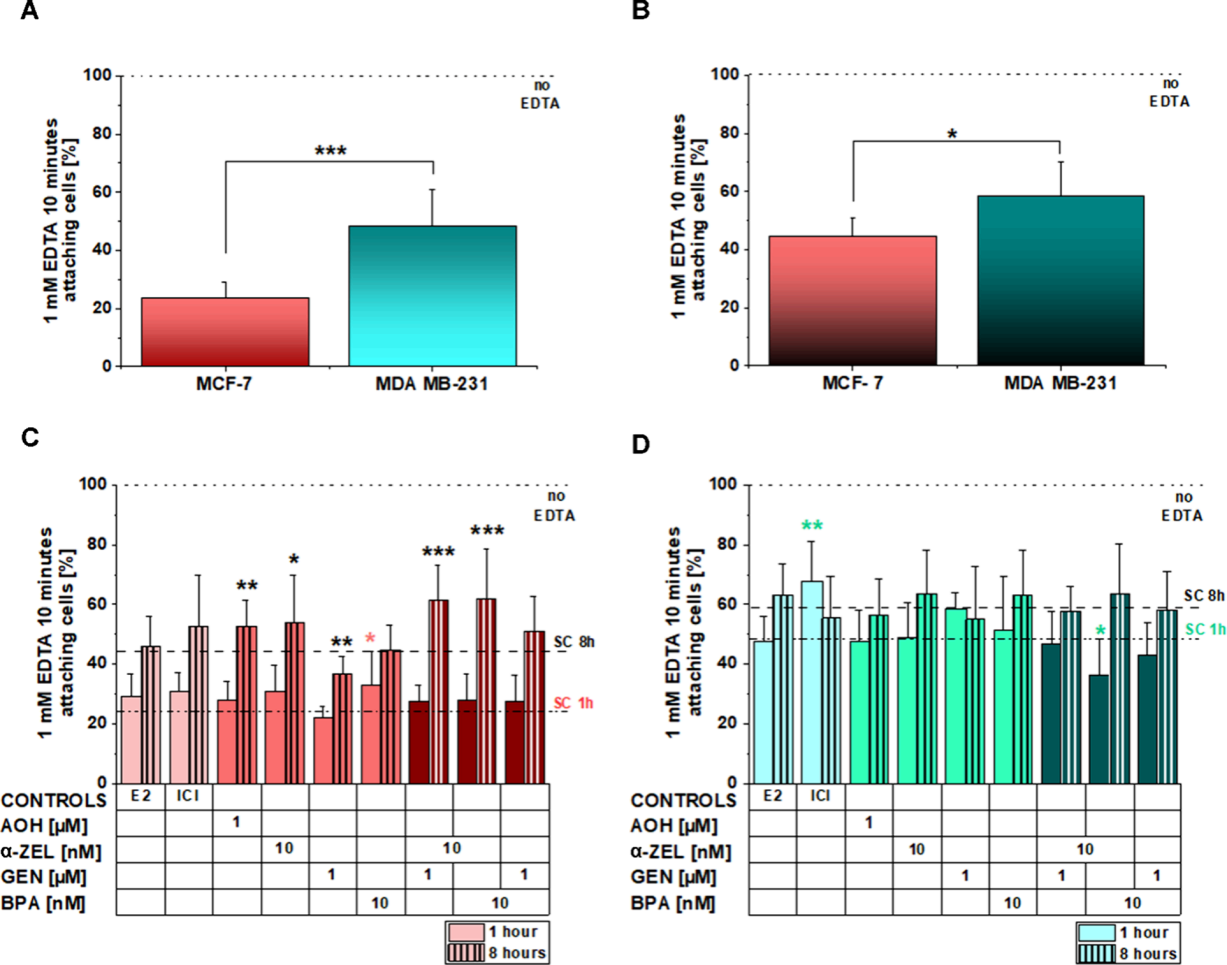
Cell attachment after performance of the deadhesion assay using 10 mM EDTA (10′). Upper panels show the behavior of MCF-7 and MDA MB-231 after (A) 1 h and (B) 8 h of hormone starvation. Bar diagrams depict the (C) MCF-7 attaching cells after 1 h (full bars) and 8 h (striped bars) of substance exposure and detachment applying EDTA and (D) MDA MB-231 cells attaching after 1 h (full bars) and 8 h (striped bars) of substance exposure and subsequent detachment. “Attaching cells” are calculated as percentage of solvent control cells not exposed to EDTA detachment solution (DS) yet incubated with EM for the same time frame as the incubation conditions (*n* > 3). Significant differences in comparison to the behavior of the solvent control (SC; dotted lines for 1 and 8 h) were calculated applying Student’s *t*-test, and indicated as *(*p* < 0.05), **(*p* < 0.01), and ***(*p* < 0.001). Colored symbols are used to mark the differences with SC after 1 h incubation and black symbols for comparison to SC after 8 h incubation.

Dissociation from the tissue constraints and detachment from the ECM are crucial steps required for independent single cancer cell motility. Building on this, the capacity of the selected xenoestrogens to modify the adhesion properties of breast cancer cells (deadhesion assay) was tested. Following 1 h of hormone starvation, namely, incubation in EM, and subsequent treatment with 1 mM EDTA, only 23% of MCF-7 cells remained attached to the cell culture wells (Figure 3A). Eight hours of hormonal deprivation enhanced the MCF-7 adhesion, resulting in an average of 44% of cells still adherent after treatment with EDTA (Figure 3B). The adaptive response of MDA MB-231 to the medium change was less pronounced. After 1 h, 48% of cells remained attached and 58% after 8 h incubation (Figure 3A,B). Aligned to the behavior of the controls, MCF-7 cells were more sensitive to exposure to the chemicals with hormonal mimicry potential (Figure 3C). For comparison, E2 and estrogen receptor antagonist ICI were included in the experimental layout and sustained an increased adhesion trend, however, without a significant effect (Figure 3C). Of note, treatment with E2 and ICI reduced cell mass/adhesion even without EDTA treatment (Supplementary Figure 1C), possibly influencing the outcome of the assay. As far as the other substances are concerned, 1 h of incubation with 10 nM BPA significantly enhanced the adhesion capacity of MCF-7 (Figure 3C). Similarly, 8 h of incubation with the mycoestrogens AOH (1 μM) and α-ZEL (10 nM) reduced the number of cells detaching after the application of EDTA. On the other hand, exposure to 1 μM isoflavone GEN rather hampered cell adhesion. The binary combination of α-ZEL + GEN as well as α-ZEL + BPA aligned with the response to α-ZEL and significantly enhanced the number of cells remaining attached to the incubation vessels (8 h of incubation, Figure 3C). In agreement with the observation that MDA MB-231 were more adherent than MCF-7, they also displayed limited sensitivity to the treatments; the adhesion behavior of MDA MB-231 was modified after 1 h exposure to ICI 1 μM, leading to enhanced adhesion of the cells and after 1 h incubation with α-ZEL + BPA with a decrease of adhesion in comparison to controls (Figure 3D). No changes were observed after 8 h of exposure (Figure 3D).

**Figure 4:**
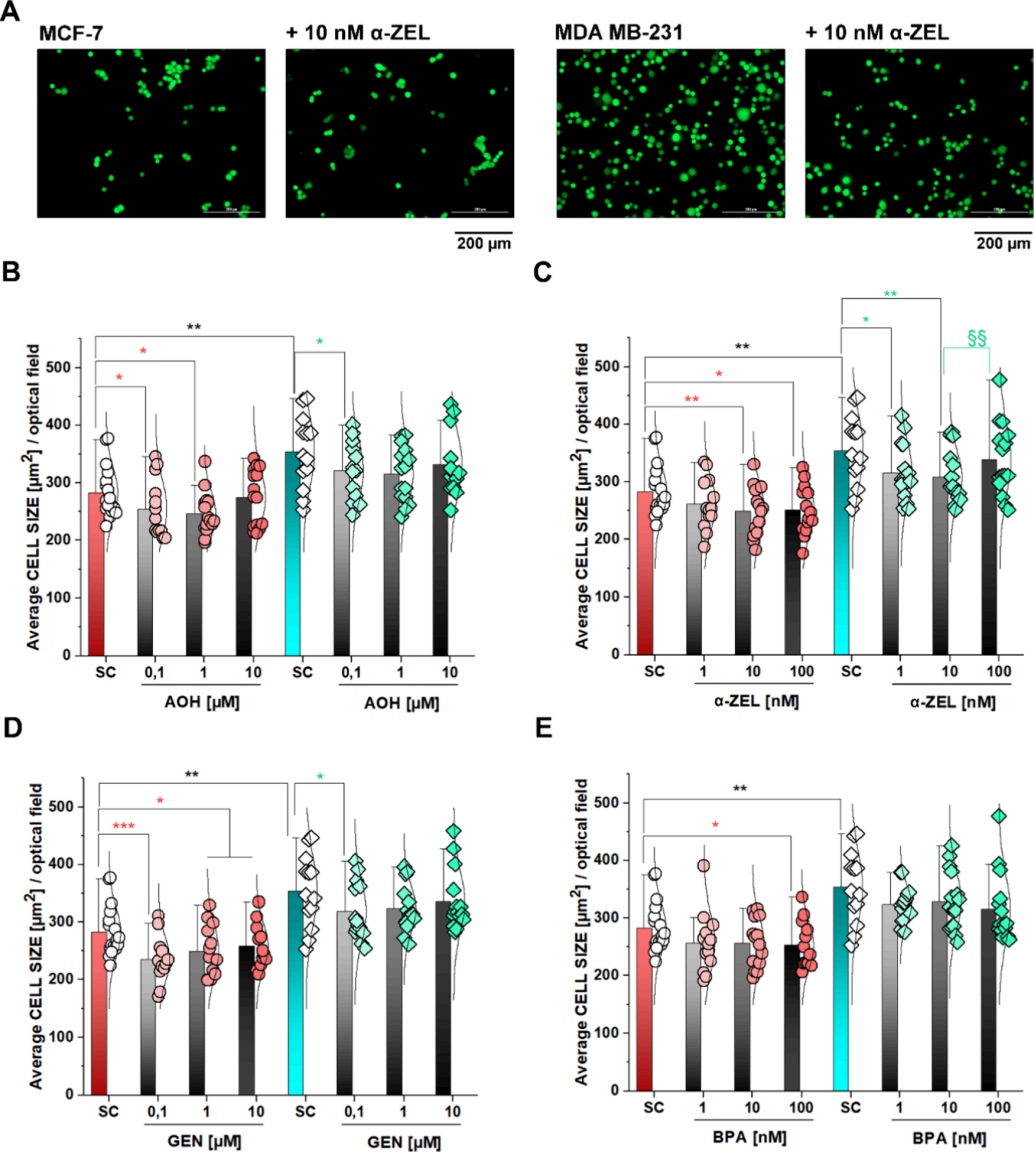
Cell adhesion/spread after 1 h settling time. (A) Appearance of the MCF-7 and MDA MB-231 cells in control conditions and after incubation with 10 nM α-ZEL (scale bars stand for 200 μm). Quantification of the average cell area/optical field upon incubation with (B) AOH, (C) α-ZEL, (D) GEN, and (E) BPA. Data are obtained after the quantification of *n* ≥ 12 optical fields from *n* = 3 independent cell preparations (biological replicates). Data groups were compared with one-way ANOVA plus the Fisher LSD test. * indicates significant difference in comparison to solvent controls, *(*p* < 0.05), **(*p* < 0.01), and ***(*p* < 0.001). § indicates significant difference within a treatment group §§ (*p* < 0.01). Treatments were performed in parallel and organized in substance-specific graphs; for visualization purposes, solvent controls (SC; MCF-7 and MDA MB-231) were repeated in all panels. Black symbols refer to the comparison between the controls of the two cell lines, orange refers to the comparisons within the MCF-7 cell line, and teal refers to the comparisons within MDA MB-231.

To shed further light on the concentration dependence of the observed effects, an adhesion-spread assay was performed (Figure 4). After 1 h settling time in control conditions or in the presence of the compounds of interest, measurement of average cell areas revealed that the MDA MB-231 had a significantly higher spread in comparison to MCF-7 (Figure 4A–E). Exposure to the xenoestrogens generally reduced the average cell size (Figure 4A–E). In line with the results obtained with the detachment assay, most regulatory events pertained to the MCF-7 cell line (Figure 4B–E). Here α-ZEL and BPA induced a concentration-dependent effect with cell shrinkage increasing parallel to the compound concentrations (Figure 4C,E). For MDA MB-231, no effect could be seen for BPA, whereas α-ZEL triggered a significant reduction of cell size when incubated at concentrations of 1 and 10 nM (Figure 4C,E). AOH and GEN seemed most effective at the lower concentrations tested for both cell lines (Figure 4B,D). Only in MCF-7, GEN maintained its effectiveness throughout the complete range of concentrations tested (0.1–10 μM; Figure 4D).

### 3.3 Effect of Dietary Xenoestrogens on Breast Cancer Cell Morphology

**Figure 5:**
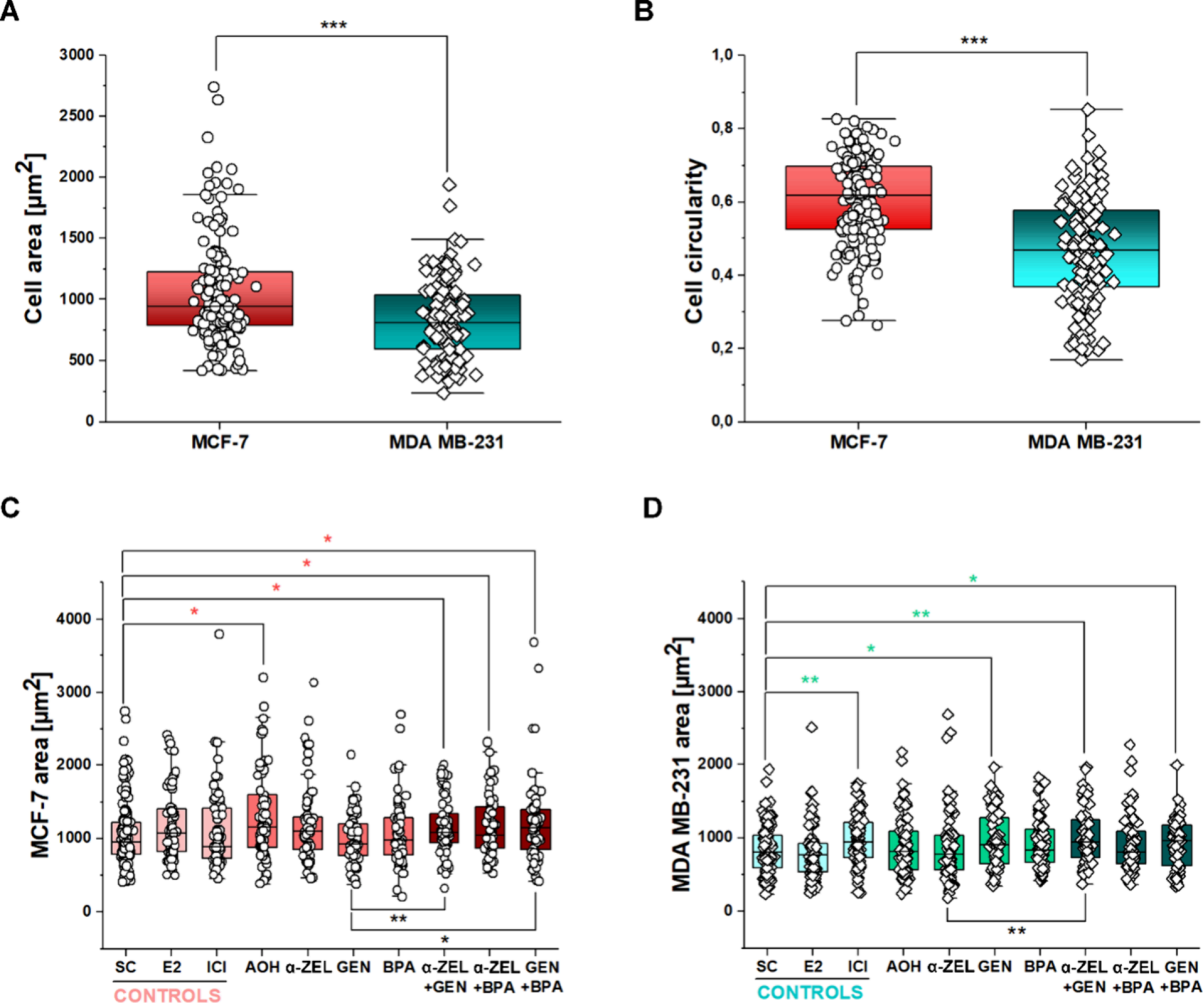
Quantification of cellular appearance after 8 h of incubation for controls (SC: solvent controls, E2 and ICI), single substance treatments (1 μM AOH, 10 nM α-ZEL, 1 μM GEN, and 10 nM BPA), and mixtures (10 nM α-ZEL + 1 μM GEN; 10 nM α-ZEL + 10 nM BPA; 1 μM GEN + 10 nM BPA). Box plots depict the distribution of (A) cell area and (B) circularity of MCF-7 vs MDA MB-231 for control conditions. (C, D) Impact of endocrine disruptive substances on the area of MCF-7 and MDA MB-231. Significant differences in comparison to the respective solvent control (colored *) or between single treatments and mixtures (black *) were determined by applying the Mann–Whitney test *(*p* < 0.05), **(*p* < 0.01), and ***(*p* < 0.001). Data result from three independent cell preparations and *n* ≥ 60 cells. Data were obtained from three independent cell preparations, quantifying *n* ≥ 60 cells for each experimental condition.

**Figure 6:**
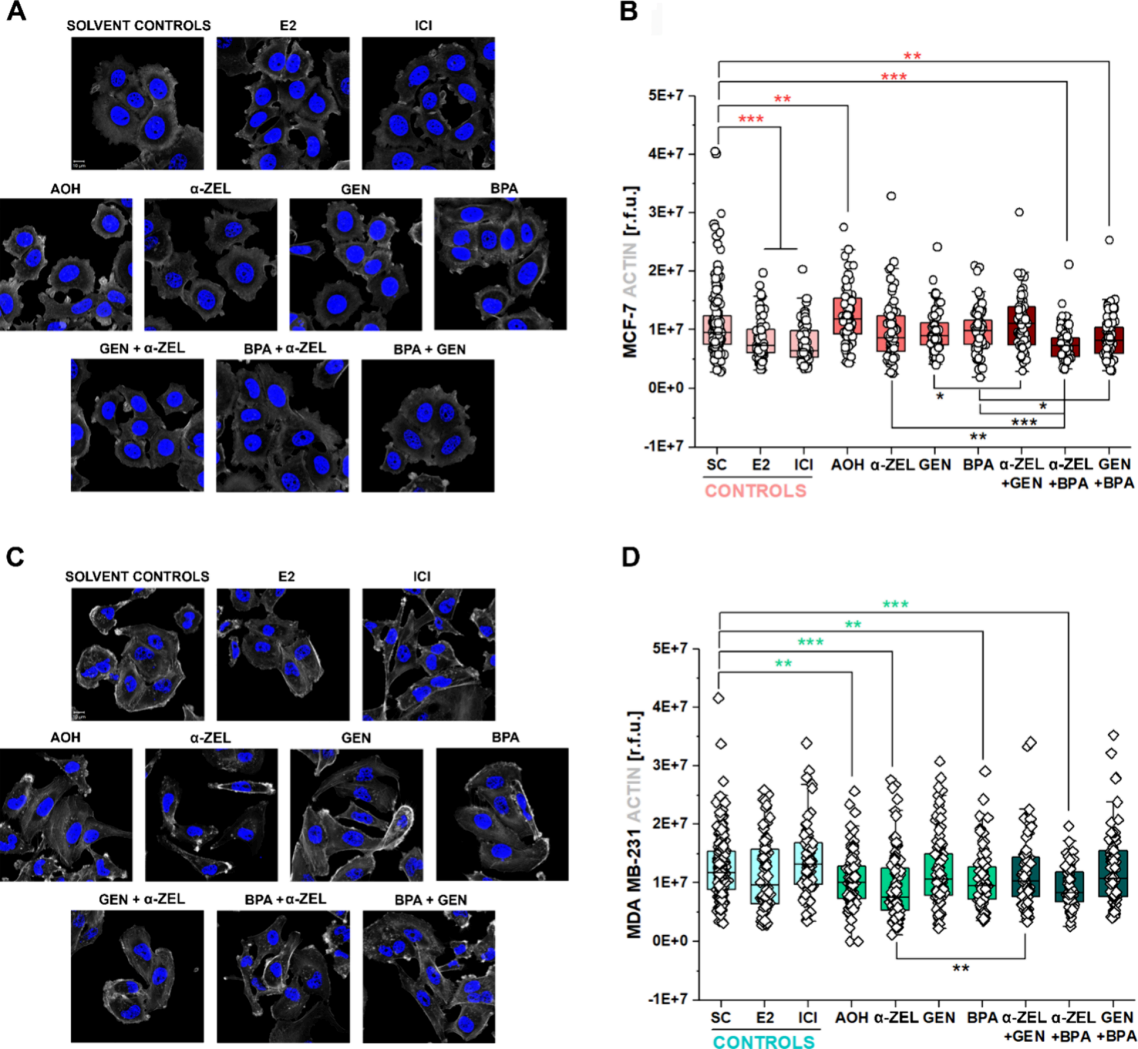
Laser confocal microscopy images of actin (white). Cell nuclei are marked in blue (DAPI staining). (A) Representative images of MCF-7 after 8 h of incubation and (B) box plots depicting image fluorescence quantification expressed as relative fluorescence units (r.f.u.). (C) Representative images of MDA MB-231 after 8 h of incubation and (D) box plots depicting image fluorescence quantification expressed as relative fluorescence units (r.f.u.). Scale bars are 10 μm. Treatments include solvent controls (SC), E2 (1 nM), ICI (1 μM), AOH (1 μM), α-ZEL (10 nM), GEN (1 μM), BPA (10 nM), and the binary mixtures α-ZEL + GEN, α-ZEL + BPA, and GEN + BPA. Significant differences in comparison to the respective solvent control (colored *) or between single treatments and mixtures (black *) were determined by the applying Mann–Whitney test *(*p* < 0.05), **(*p* < 0.01), and ***(*p* < 0.001). Data result from three independent cell preparations and *n* ≥ 60 cells.

To verify if the exposure to the xenoestrogens could affect cell morphology and cytoskeletal apparatus, morphometric descriptors (area, circularity, Figure 5) and actin signal intensity (Figure 6) were measured. MCF-7 cells had, on average, a bigger area and more circular appearance in comparison to MDA MB-231 (Figure 5A,B). Exposure to 1 μM AOH and to the binary mixtures α-ZEL + GEN, α-ZEL + BPA, and GEN + BPA significantly increased the area of MCF-7 cells in comparison to that of controls (Figure 5C). For the binary mixtures containing GEN (α-ZEL + GEN and GEN + BPA) the obtained values were also significantly higher in comparison to those of the isoflavone alone (Figure 5C). In comparison, the cell area increased for MDA MB-231 after incubation with 1 μM ICI or GEN-including treatments (1 μM GEN; α-ZEL + GEN and GEN + BPA, Figure 5D). None of the incubation conditions significantly altered cell circularity for either of the breast cancer cell lines (Supplementary Figure 3). As far as actin was concerned, in both cell types, the tested compounds modified the appearance of the cytoskeletal element. In MCF-7, ER agonist E2 (1 nM) as well as ER antagonist ICI (1 μM) reduced the actin network signal (Figure 6A,B). A similar response was observed also for two binary mixtures containing BPA (α-ZEL + BPA, and GEN + BPA, Figure 6B), whose values were also lower than BPA as a single molecule (Figure 6B). In contrast, AOH (1 μM) significantly enhanced the actin fluorescence signal (Figure 6A,B). In MDA MB-231, actin fluorescence was reduced by incubation with single substances AOH, α-ZEL, and BPA, as well as the binary mixture of α-ZEL + BPA (Figure 6C,D).

### 3.4 Effect of Dietary Xenoestrogens on Integrin β1 in Breast Cancer Cells

**Figure 7:**
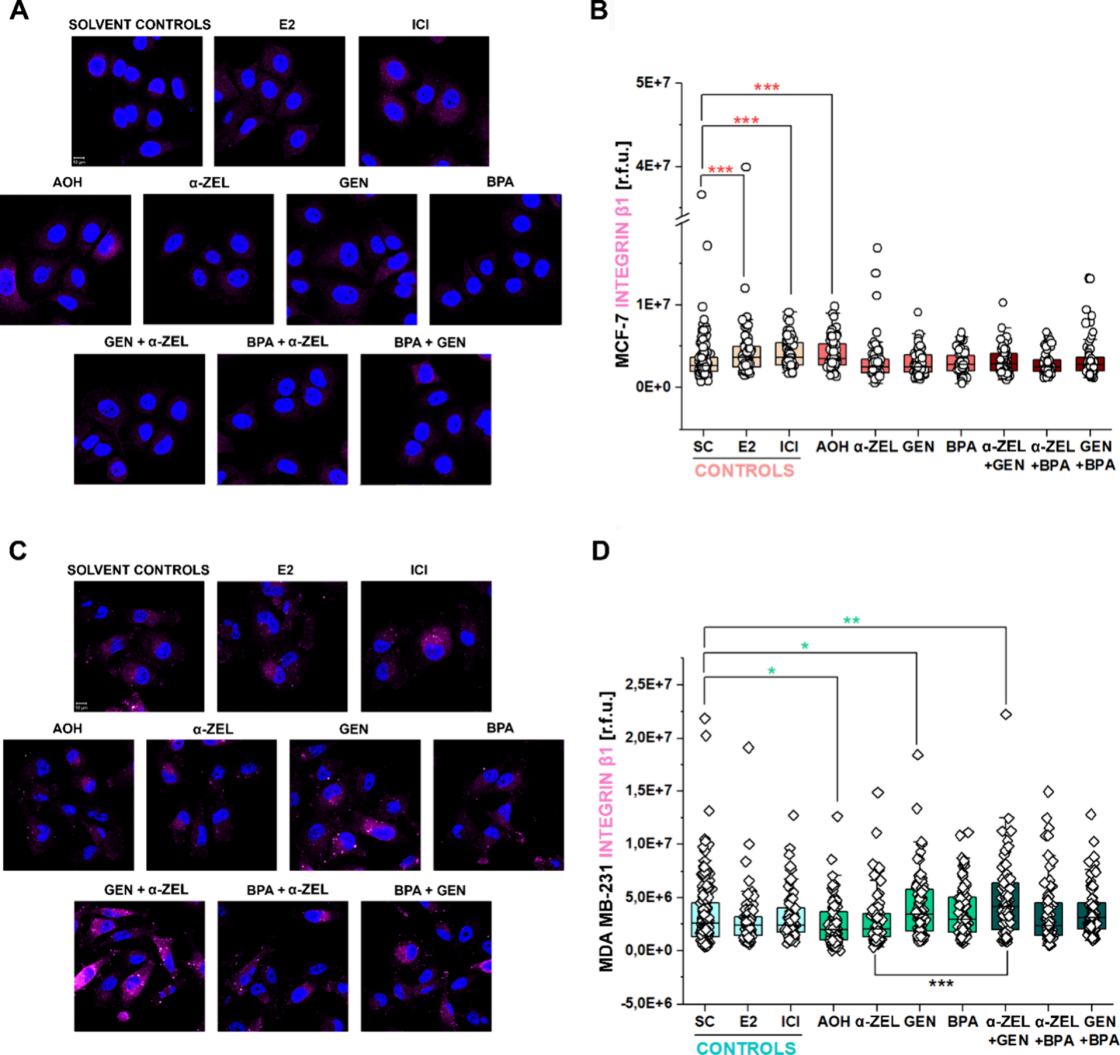
Laser confocal microscopy images of the integrin β1 protein (magenta). Cell nuclei are marked in blue (DAPI staining). (A) Representative images of MCF-7 after 8 h incubation and (B) box plots depicting image fluorescence quantification expressed as relative fluorescence units (r.f.u.). (C) Representative images of MDA MB-231 after 8 h incubation and (D) box plots depicting image fluorescence quantification expressed as relative fluorescence units (r.f.u.). Scale bars stand for 10 μm. Treatments include solvent controls (SC), E2 (1 nM), ICI (1 μM), AOH (1 μM), α-ZEL (10 nM), GEN (1 μM), BPA (10 nM), and the binary mixtures α-ZEL + GEN, α-ZEL + BPA, and GEN + BPA. Significant differences in comparison to the respective solvent control (colored *) or between single treatments and mixtures (black *) were determined by applying the Mann–Whitney test *(*p* < 0.05), **(*p* < 0.01), and ***(*p* < 0.001). Data result from three independent cell preparations and *n* ≥ 60 cells.

Following the working hypothesis that xenoestrogens could interact with the adhesion proteins, we examined if exposure to the compounds of interest could reflect the distribution and intensity levels of activated integrin β1 by immunofluorescence. In MCF-7, both E2 and ICI, as well as mycotoxin AOH, increased the detection of integrin β1 (Figure 7A,B). For MDA MB-231 in turn, exposure to AOH led to a reduced signal for the integrin β1 protein. Incubation with GEN as well as with the binary mixture α-ZEL + GEN enhanced the detection of activated integrin β1 (Figure 7C,D). In this case, values obtained upon incubation with α-ZEL + GEN were also significantly higher in comparison to the application of α-ZEL as a single compound (Figure 7C,D).

### 3.5 Effect of Dietary Xenoestrogens on Intracellular Cascades Relevant for Cell Adhesion and Motility

**Figure 8:**
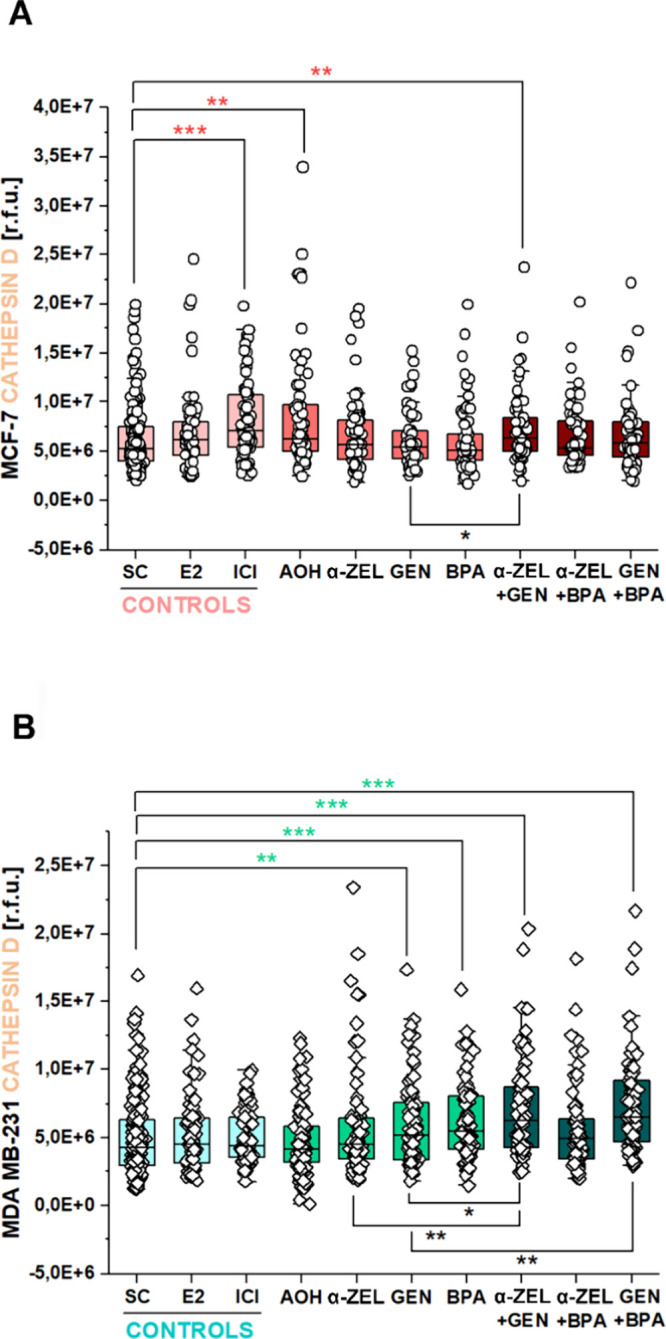
Quantification of cathepsin D expressed as relative fluorescence units (r.f.u.) for (A) MCF-7 and (B) MDA MB-231. Treatments include solvent controls (SC), E2 (1 nM), ICI (1 μM), AOH (1 μM), α-ZEL (10 nM), GEN (1 μM), BPA (10 nM), and the binary mixtures α-ZEL + GEN, α-ZEL + BPA, and GEN + BPA. Significant differences in comparison to the respective solvent control (colored *) or between single treatments and mixtures (black *) were determined by applying the Mann–Whitney test *(*p* < 0.05), **(*p* < 0.01), and ***(*p* < 0.001). Data result from three independent cell preparations and *n* ≥ 60 cells.

To evaluate additional aspects relevant for intracellular signaling cascades in the regulation of breast cancer cell morphology and motility, the role of cathepsin D was investigated. Cathepsin D is an estrogen-regulated^55^ intracellular protease that can contribute to regulating actin dynamics^56^ and whose role was previously discussed in metastatic breast cancer.^57^ Indeed, for MCF-7, the ER antagonist ICI, as well as AOH, significantly enhanced the immunofluorescence signal of cathepsin D. Moreover, incubation with the binary mixture of α-ZEL + GEN returned higher signal intensity values in comparison to solvent controls and in comparison to GEN (Figure 8A and Supplementary Figure 4). For MDA MB-231 cells, the plasticizer BPA and the isoflavone GEN significantly enhanced the immunofluorescence signal of cathepsin D. This response was maintained also in the binary mixtures containing genistein, namely, α-ZEL + GEN and GEN + BPA, which returned values even higher than the application of GEN or α-ZEL alone (Figure 8B and Supplementary Figure 4).

### 3.6 *In Silico* Analysis of the Interaction of Dietary Xenoestrogens with Integrins

**Figure 9:**
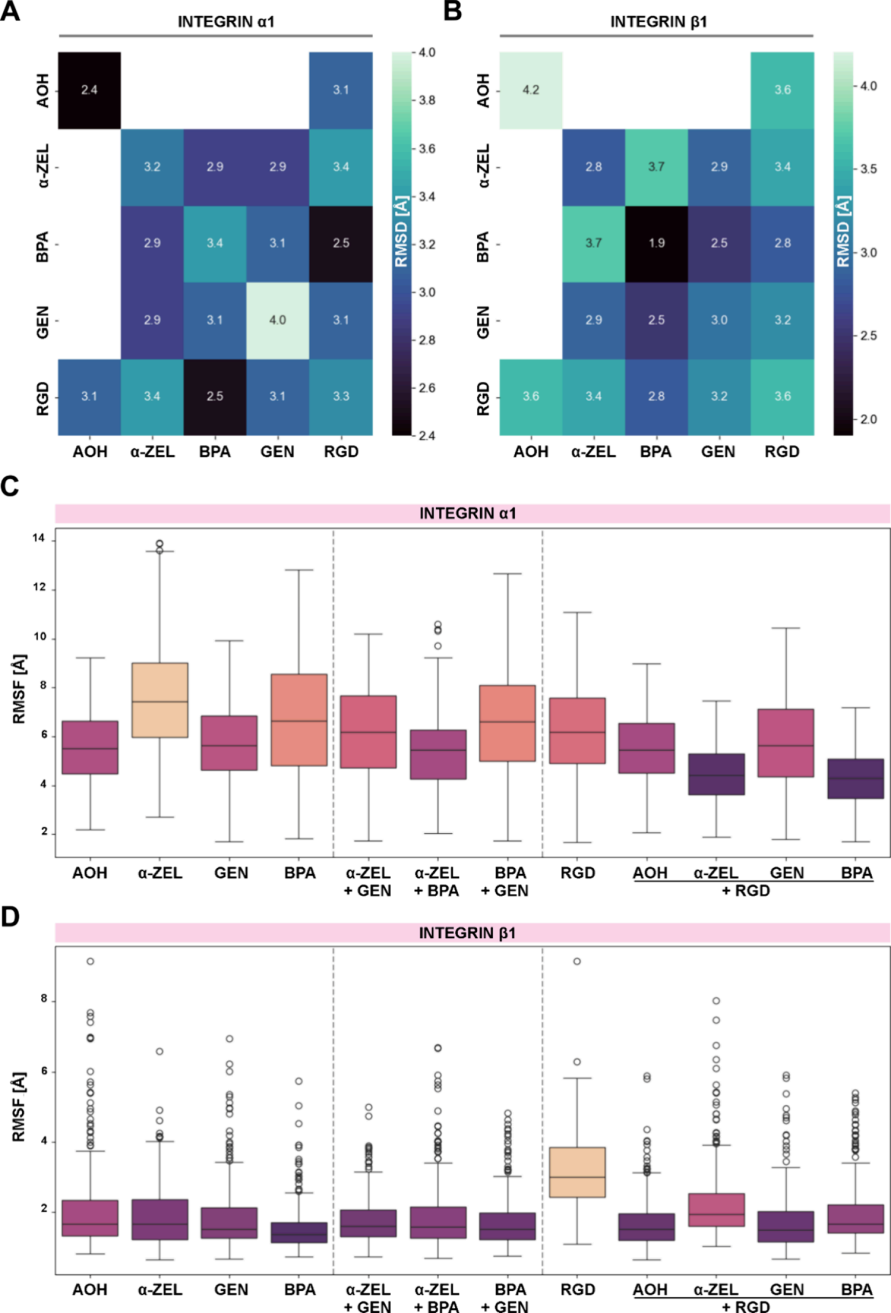
*In silico* evaluation of the interaction between the xenoestrogens and integrin subunits α1 and β1 in the presence or absence of RGD. Heatmaps depicting the root-mean-squared deviation (RMSD) values for the integrin (A) α1 and (B) β1. Boxplots of the RMSF values of the amino acids belonging to (C) α1 and (D) β1. Colors are representative of the stability of the interactions with violet (more stable) to orange (less stable).

To deepen the hypothesis that xenoestrogens could serve as binding partners for integrins, *in silico* analyses were performed. In this case, the integrin binding peptide RGD^58^ was included in the experimental layout as a reference molecule (Figure 9). The simulation of integrin in the absence of any chemical led to partial unfolding of its domains, rendering the respective results inconsequential. Figure 9 displays the root-mean-square deviation (RMSD; Figure 9A,B) and fluctuation (RMSF; Figure 9C,D; Supplementary Figure 5) of the protein domains during the 200 ns simulation, indicating its overall stability. The diagonal elements of the heatmap in Figure 9A,B represent simulations containing only one of the compounds, namely, AOH, α-ZEL, BPA, GEN, and RGD in the protein simulation, whereas off-diagonal values refer to the corresponding mixtures. For the RMSD data, the darker the area is depicted in the heatmap, the more stable the integrin α1 and β1 domains are when in contact with the compounds. For the integrin α1, the presence of GEN returned the highest RMSD value (4.0; Figure 9A) and AOH the lowest (2.4; Figure 9A), similar to the RGD + BPA combination (2.5; Figure 9A). The highest RMSD value for integrin β1 was calculated in the presence of AOH (4.2; Figure 9B) and the lowest for BPA (1.9; Figure 9B). Interestingly, α1 and β1 domains mostly exhibited opposite trends for the stabilization and destabilization when analyzing the data derived from the mixtures. For GEN and BPA adding a second compound seems to stabilize the α1 domain. In the case of BPA, the β1 domain is also destabilized. The trend is opposite for AOH. With regard to α-ZEL, all additional compounds destabilize the β1 domain. Since RGD is reported to bind to the integrin β1 subunit,^59^ adding this compound in the mixtures destabilizes the protein in comparison to single xenoestrogens, except for the combination AOH + RGD. For the α1 domain, such a clear trend was not observed. Figure 9C,D displays box plots of the RMSF values for the residues belonging to the α1 (Figure 9C) and β1 (Figure 9D) domain. The left, middle, and right columns relate to the single xenoestrogen compounds, the xenoestrogen mixtures, and the RGD mixtures, respectively. In general, the RMSF of the α1 domain is higher than that of the β1 domain. Pertaining to the β1 domain, only RGD returned higher fluctuations than all other systems. The outliers in Figure 9D indicate that particular residues of the β1 domain interact with the compounds resulting in much higher RMSF values than the average (see Supplementary Figure 5). In contrast, the RMSF values related to the α1 residues do not display residue specific behavior, yielding fewer outliers than for the residues in β1.

To alter the protein stability, the compounds of interest must interact with the amino acids of the protein. The interaction presupposes a contact with a particular amino acid *i*, which can be measured by the average contact number ⟨*C*_*i*_⟩
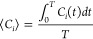
derived from the transient contact number *C*_*i*_(*t*) at time step *t*, which counts the chemicals with a center-of-mass distance of less than 0.7 nm to the center-of-mass of the amino acid *i*. The contact numbers are evaluated at intervals of 100 ps and averaged over the simulation period *T*. Observations indicate that AOH, α-ZEL, BPA, GEN, and RGD tend to have a greater-than-average number of contacts with arginine, glutamine, alanine, and (iso)leucine. The fact that these amino acids represent charged and hydrophobic amino acids suggests that Coulombic or dipolar interactions do not enforce the contacts. Rather, the local shape and roughness of the protein surface dictate the way the chemical binds to the amino acids.

**Figure 10:**
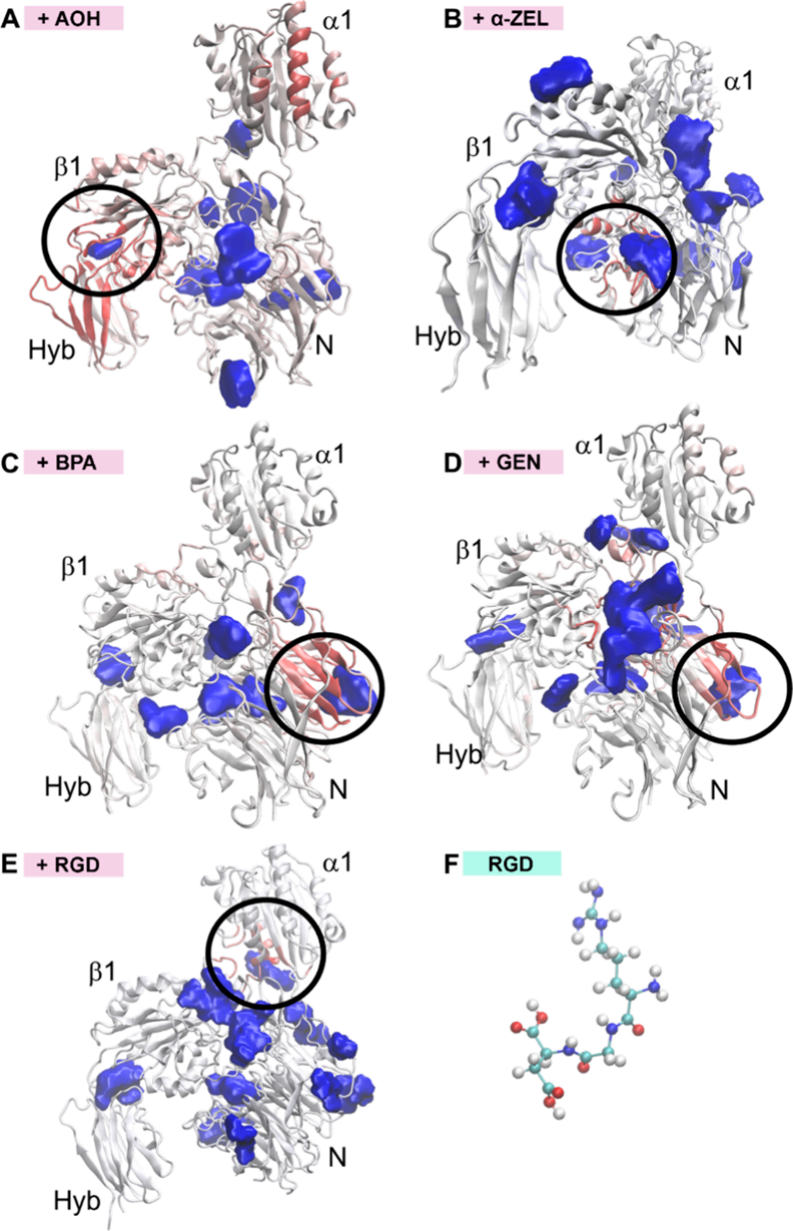
Comparison of the favorable spots detected by docking (blue areas) using SWISSDOCK and by the average contact number (red parts of the protein) derived from MD simulations for integrin α1 and β1 subunits. The black circles indicate the best consensus between these two methods for (A) AOH, (B) α-ZEL, (C) BPA, (D) GEN, and (E) RGD. (F) Structure of RGD peptide.

By replacing the B-factor data of the protein PDB file with the average contacts ⟨*C*_*i*_⟩, one may visualize the most frequently visited spots on the protein surface, as shown in Figure 10 (red-colored secondary structures). Notably, the docking program detected more binding sites (blue areas) than the contact analysis of the MD trajectory. Except for AOH, neither SWISSDOCK nor the contact analysis detected very favorable spots in the α1 domain of the protein. Most of the preferred sites in the MD simulations are located at the N-domain of the protein. In the case of AOH, the best consensus between SWISSDOCK and the MD simulations concerns the docking area between the β1 and the Hyb domain (see the black circle in Figure 10A). The most frequently visited sites for α-ZEL are in the N-domain near the β1 and Hyb domains (black circle in Figure 10B). BPA and GEN prefer the same remote side of the N-domain, which was also detected by SWISSDOCK (Figure 10C,D). In the case of GEN, additional agreement for a docking site exists at the border between the α1 and the β1 domain. The behavior of RGD (Figure 10E) and RGD peptide structure (Figure 10F) is included for comparison.

The competition between the chemicals for the protein domain interaction can be inferred by analyzing the domain contact number *C*_domain_:
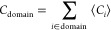
which is simply the sum of the average contact numbers of their amino acids. As a chemical may have multiple contacts simultaneously, *C*_domain_ may exceed the total number of the chemicals present in the simulation, as evidenced in Supplementary Table 1. This observation reinforces the significance of the protein surface shape and roughness in the process of ligand binding. As demonstrated in Table 1, the domain contact numbers for the α1 and β1 domains were relatively low when only BPA was present in the solution, with recorded values of 9.1 and 7.8, respectively. However, the addition of α-ZEL, GEN, or RGD to the mixture resulted in a significant increase in contact numbers, with values of 53.5/51.2/20.9 for the α1 domain and 18.9/20.5/24.2 for the β1 domain. It is worth noting that the domain contact number may also be decreased by introducing certain compound combinations. For instance, the *C*_domain_ for GEN at the β1 domain was 36.7; however, upon the addition of α-ZEL, BPA, or RGD, this value decreased significantly. It should be noted that this change led to an increase in the number of contacts of GEN with the α1 domain. Therefore, α-ZEL, BPA, and RGD effectively expel GEN from the β1 domain and relocates it to the α1 domain. Similar behavior could be observed also for AOH and GEN when combined with RGD. In this case, the presence of the peptide seems to displace the test compounds from the β1 subunit and increase the number of contacts in the α1 domain.

## 4 Discussion

Metastases play a major role in tumor-related mortality.^60,61^ However, considering the complexity of the metastatic cascade, combined epidemiological and mechanistic studies are needed to identify the causal relationships. For dietary and environmental contaminants, studies elucidating molecular mechanisms are essential to sustain hazard characterization and, by doing so, support risk assessment. In line with the working hypothesis of this article, it was postulated that xenoestrogens could affect crucial steps necessary for the formation of the metastatic clones, particularly by inducing alterations of the cell cytoskeleton or by serving as binding partners for adhesion molecules like integrins. During metastases formation, the cells constituting the primary tumor progressively change toward a mesenchymal phenotype (epithelial-to-mesenchymal transition, EMT). EMT promotes the separation of cells from the primary tumor site and eases the spread toward secondary sites.^61^ It is commonly acknowledged that these steps imply changes in the cellular structure and biophysical properties.^62−64^ Not surprisingly, the ability of cancer cells to alter their cytoskeleton is one of the crucial characteristics to initiate the metastatic process.^65^ At the molecular level, this is mediated by several players; integrins, for example, serve as anchoring units, enabling the adhesion to the ECM on the one side but also the development of traction forces necessary for cell motility.^66−68^ Considering the central role of cell motility and mechanotransduction in cancer development,^69−72^ the involvement of integrins in breast cancer progression has been extensively studied.^73^ However, the detrimental effect of endocrine disruptive molecules in this respect has only recently started to emerge. Previous studies described the capability of BPA to enhance collective cell migration (measured by the wound healing assay) in several cancer cell lines including MDA MB-231 and to activate integrin β1.^74^ BPA was also reported to increase adhesion in HUVEC cells^75^ and to reduce migration and expression of integrin α5 and β1 in human primary extravillous trophoblast cells.^76^ Here, it was possible to expand these observations to complementary assays for two cell lines and to a broader spectrum of molecules. Additionally, moving toward real-life exposure scenarios, compound combinations were included in the evaluation, and regulatory events could be observed in short-term assays (1 h) at concentrations that are not cytotoxic (i.e., Figures 3 and 4 and Supplementary Figure 1). Importantly, this approach demonstrated how the exposure to the same molecules can be associated with different outcomes according to the cell line investigated, which, in more general terms, might suggest caution in the interpretation of the data sets generated with only one cell line. Across the different assays, coherent responses could be observed in the behaviors of the MCF-7 and MDA MB-231 cells. For example, 1 h hormonal deprivation (experimental medium, EM) returned the lowest adhesion values for MCF-7 after EDTA treatment (Figure 3A). Increasing exposure to EM to 8 h returned almost double cell attachment in comparison to 1 h (Figure 3A,B). Supporting the results obtained in the deadhesion assay, incubation with estrogen antagonist ICI significantly increased integrins expression in MCF-7 (Figure 7B). Also considering the response of integrin β1 to E2 (Figure 7B) this generally aligns with the working hypothesis of the manuscript, namely, supports the presence of a crosstalk between hormones/hormonal mimicry agents and the activity of the adhesion proteins. Along these lines, following 24 h of hormonal reduction in EM, exposure of MCF-7 to the tested compounds returned most regulatory events in the modulation of cell spread/adhesion (Figure 4B–E). This infers for a high adaptive plasticity of MCF-7 in response to changes of the hormonal/xenoestrogens profile of the extracellular medium, which would also agree with the differential expression of hormonal receptors in comparison to the MDA MB-231.^34^ Reading across the data, incubation with ICI was not associated with morphometric adaption in MCF-7 (Figure 5C) despite increasing the intensity of the integrin β1 signal (Figure 7A,B). In this regard, a parallel increase in the protease cathepsin D and a decrease in actin density (Figures 6B and 8A) infer for a loss of cytoskeletal integrity and most probably account for the incapacity of structural accommodation. A similar, albeit less pronounced, phenotype could be observed also for incubation with E2; here, lack of variation in cell morphology in MCF-7 (Figure 5C) was accompanied by decreased actin signal (Figure 6B) increased integrin signal (Figure 7B) and a trend toward increase in cathepsin D signal (Figure 8A). In line with this interpretation, incubation with ICI was not effective on cathepsin D or actin in MDA MB-231 cells; in this case, application of the ER antagonist significantly increased cell adhesion (1 h of incubation, Figure 3D) and cell area (Figure 5D). These observations seem in good agreement with literature describing the expression of cathepsin D enhanced by E2 in MCF-7 and also higher protease levels in this cell type in comparison to MDA MB-231.^55^ In the pathophysiological context, the gene expression and secretion of the aspartic protease cathepsin D were previously reported to be upregulated in metastatic breast cancer.^77^ Intriguingly, exposure to food-contaminant mycotoxin AOH significantly increased the expression of cathepsin D, similar to the E2 antagonist ICI and the combination of GEN plus α-ZEL (MCF-7, Figure 8A). Even more pronounced was the response of the highly metastatic MDA MB-231 cells, for which BPA, GEN and the combinations α-ZEL + GEN and BPA + GEN increased the protease expression (Figure 8B). Complementary to these mechanisms, ER possess extranuclear functions that contribute to the regulation of actin cytoskeleton^78−80^ therefore an involvement of these cascades in the activity profile of compounds with hormonal mimicry potential cannot be excluded.

Pertaining the role of integrin β1, several incubation conditions returned significant variations in the immunolocalization of the adhesion protein (Figure 7). These findings also aligned with the *in silico* analysis, hence molecular dynamics trajectories returned increased RMSF values for the integrin α1 domain, indicating its destabilization (Figure 9C; Supplementary Figure 5). The comparison between docking and molecular dynamics simulations of the protein sites most frequently visited by the molecules returned AOH binding affinity on both α1 and β1 subunits (Figure 10A). This is in good agreement with the immunofluorescence data, which returned significant variation of the integrin expression levels in cells treated with the mycotoxin (Figure 7B,D). These results could also contribute to explaining the outcome of the adhesion/spread assay, where higher concentrations of AOH (10 μM; Figure 4B) returned cell areas comparable to controls and no reduction as for the lowest concentrations tested (0.1–1 μM; Figure 4B). Obviously, other molecular mechanisms cannot be excluded, i.e. nonmonotonic dose response (NMDR) behaviors previously described for some environmental endocrine disruptive chemicals.^81^ In addition, *in silico* analysis revealed that BPA and GEN display a similar affinity for the N-domain (Figure 10C,D). This is in good agreement with the observation that when the two compounds are combined experimentally with α-ZEL they return a similar activity profile with enhanced adhesion (MCF-7, Figure 3C) and decreased migration (MCF-7, Figure 2C). In turn, this synergy appears to be reduced when the two compounds are combined, which is coherent with the notion that BPA could effectively displace GEN from its binding site (Table 1). Along this line, in MDA MB-231 the increase in the migration speed triggered by GEN was reduced by co-application of BPA (Figure 2D). However, supportive of the differential response among the cell lines, in MDA MB-231, the combination of α-ZEL + GEN increased integrin β1 immunolocalization (Figure 7C,D) and cell area (Figure 5D) and reduced cell migration speed in comparison to GEN alone (Figure 2D). α-ZEL + BPA, in turn, had no effect on the integrin β1 signal (Figure 7D), but rather decreased actin intensity (Figure 6D) and cell adhesion (Figure 3D).

In summary, it was possible to demonstrate that xenoestrogens significantly modify the morphology and motility of breast cancer cells *in vitro* after short-term exposure. Even with the clear limitations of the *in vitro*/*in silico* systems, the breast cancer cells used in this study are often utilized as models representing different stages of the disease. Here, MCF-7 and MDA MB-231 responded differentially to stimulation with hormonal mimicry agents. Being that the experimental outcome is tightly related to the cell model used, this could contribute toward describing the challenges related to the translation of the experimental data obtained *in vitro* to *in vivo* scenarios. Nevertheless, the data collected in the study at hand delineate integrins as possible targets for molecules with an endocrine disrupting potential. Considering that integrins are on focus for the development of new anticancer drugs,^58^ interference of xenobiotics with these proteins would not only affect tumor pathophysiology, but also potentially modulate the activity profile of chemotherapeutic agents. Obviously, to better grasp the relevance of the experimental data with respect to disease progression, more data are needed, including further mechanistic investigations, as well as biomonitoring studies connecting disease stages to the evaluation of environmental and dietary exposures. In conclusion, this study supports the role of endocrine active chemicals in modulating cell adhesion and paves the way to a better comprehension of the contribution of xenoestrogens in breast cancer development.

## Supporting Information Available

Supplementary Figure 1: Cell viability assays; Supplementary Figure 2: Evaluation of single cell motility persistence (8 h; MCF-7 and MDA MB-231); Supplementary Figure 3: Evaluation of the circularity of MCF-7 and MDA MB-321; Supplementary Figure 4: Representative appearance of MCF-7 and MDA MB-321 after cathepsin D staining; Supplementary Figure 5: Profiles plots of the root-mean-squared fluctuation analysis; Supplementary Table 1: Composition of the molecular dynamics simulation systems (PDF)

## ABBREVIATIONS

AOH: alternariol
BPA: bisphenol A
CD-FCS: charcoal-stripped fetal calf serum
E2: 17-β-estradiol
EDCs: endocrine disruptive chemicals
EM: experimental medium
EMT: epithelial-to-mesenchymal transition
ER: estrogen receptor
GEN: genistein
MD: molecular dynamics
NOC: nocodazole
R.f.u.: relative fluorescence units
RGD: peptide sequence Arg-Gly-Asp
RMSD: root-mean-square deviation
RMSF: root-mean-square fluctuations
SRB: sulforhodamine B
α-ZEL: α-zearalenol
