# Supplementary material for: Short-Term Exposure to Foodborne Xenoestrogens Affects Breast Cancer Cell Morphology and Motility Relevant for Metastatic Behavior In Vitro
Source: Chem Res Toxicol. 2024 Sep 12;37(10):1634–50. doi: 10.1021/acs.chemrestox.4c00061 (PMC11497359; doi:10.1021/acs.chemrestox.4c00061)
Supplement: Supplementary file 1 — tx4c00061_si_001.pdf [file tx4c00061_si_001.pdf]

Supporting Information

Short-term exposure to foodborne xenoestrogens affects breast cancer cell morphology and motility relevant for metastatic behavior *in vitro*

Giorgia Del Favero [1,2]*, Janice Bergen [1,2,3], Lena Palm [4], Christian Fellinger [4,5,6], Maria Matlaeva [4], András Szabadi [4], Ana Sofia Fernandes [7], Nuno Saraiva [7], Christian Schröder [4], Doris Marko [1]

[1]Department of Food Chemistry and Toxicology, Faculty of Chemistry, University of Vienna, 1090 Vienna, Austria

[2]Core Facility Multimodal Imaging, Faculty of Chemistry, University of Vienna, 1090 Vienna, Austria

[3] Vienna Doctoral School in Chemistry (DoSChem), University of Vienna, Währinger Str. 42, 1090 Vienna, Austria

[4] Computational Biological Chemistry Department, Faculty of Chemistry, University of Vienna, 1090 Vienna, Austria

[5] Department of Pharmaceutical Sciences, Faculty of Life Sciences, University of Vienna, 1090 Vienna, Austria

[6] Christian Doppler Laboratory for Molecular Informatics in the Biosciences, Department for Pharmaceutical Sciences, University of Vienna, 1090 Vienna, Austria

[7] CBIOS, Universidade Lusófona's Research Center for Biosciences & Health Technologies, 1749-024 Lisboa, Portugal

*** Correspondence:**

* Del Favero Giorgia: giorgia.del.favero@univie.ac.at

Department of Food Chemistry and Toxicology and Core Facility Multimodal Imaging
Faculty of Chemistry, University of Vienna, 1090 Vienna, Austria

Table of contents	Pg.
**Supplementary Figure 1.** Cell viability assays.	**S3**
**Supplementary Figure 2.** Evaluation of single cell motility persistence (8h; MCF-7 and MDA MB-231).	**S4**
**Supplementary Figure 3.** Evaluation of the circularity of MCF-7 and MDA MB-321	**S5**
**Supplementary Figure 4.** Representative appearance of MCF-7 and MDA MB-321 after cathepsin D staining.	**S6**
**Supplementary Figure 5.** Profiles plots of the root-mean-squared fluctuations (RMSF) analysis.	**S7**
**Supplementary Table 1.** Composition of the molecular dynamics simulation systems	**S7**

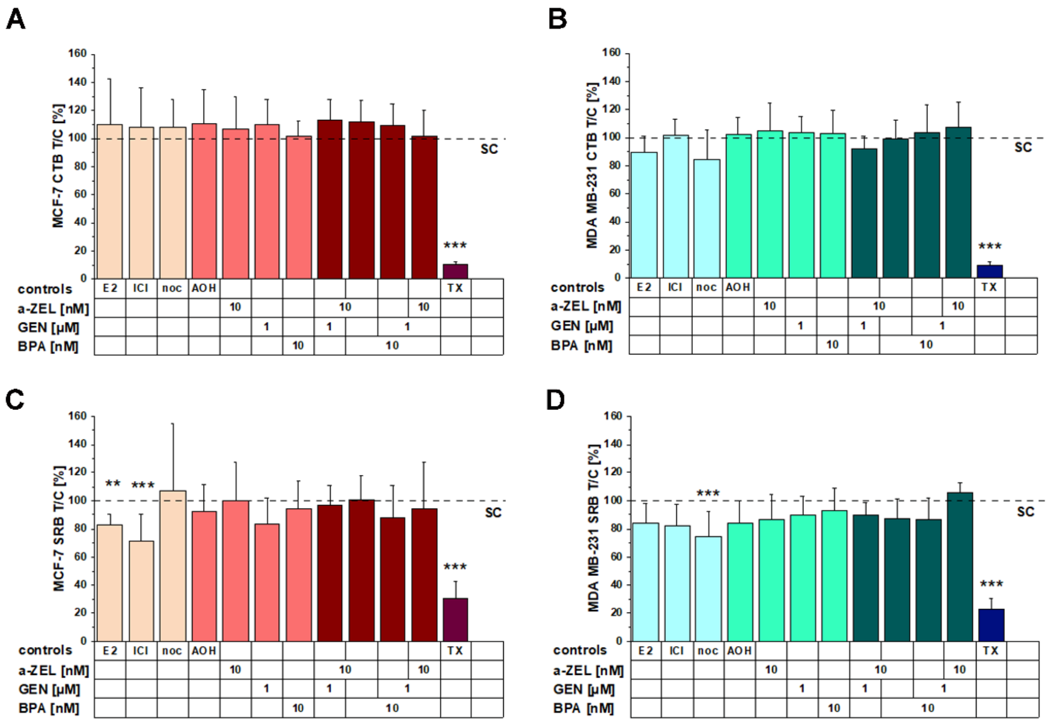

Supplementary Figure 1. Cell viability testing after eight hours of compound incubation. Cell metabolic activity was tested via CellTiter-Blue Assay (CTB) and is depicted in (A) for MCF-7 and in (B) for MDA MB-231. Cell mass /adhesion was measured via Sulforhodamine-B Assay (SRB) and is depicted in (C) for MCF-7 and in (D) for MDA MB-231. Metabolic activity as well as protein contents were normalized to the solvent control (DMSO), TritonX-100 (0.1%; TX) served as positive control. SC: solvent controls, 1 nM E2, 1µM ICI, 0.1 µM nocodazole, single substance treatments (1µM AOH, 10 nM α-ZEL, 1µM GEN and 10 nM BPA) and mixtures (10 nM α-ZEL + 1µM GEN; 10 nM α-ZEL + 10 nM BPA; 1µM GEN + 10 nM BPA; 10 nM α-ZEL + 1µM GEN + 10 nM BPA). Significant differences in comparison to the solvent controls were calculated by applying two-sample Students' *t*-test and are marked ** (p<0.01), *** (p<0.001). Experiments were performed on n ≥ 3 cell preparations and measured in technical triplicates.

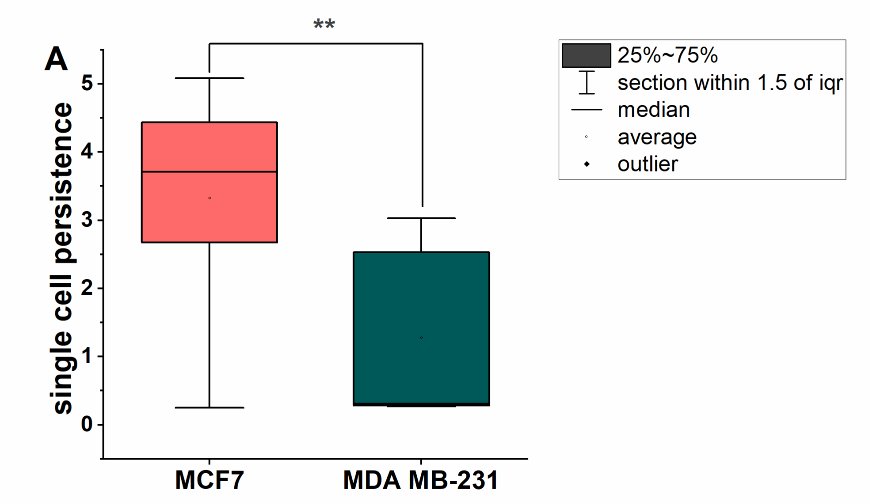

Supplementary Figure 2. Single cell persistence over 8 hours of single cell tracking. (A) shows differences in breast cancer cell line directional persistence parameter for solvent control conditions (n ≥ 270). Significant differences were determined by Mann-Whitney-Testing via comparison of median persistence values and are marked with asterisks: ** (p<0.01).

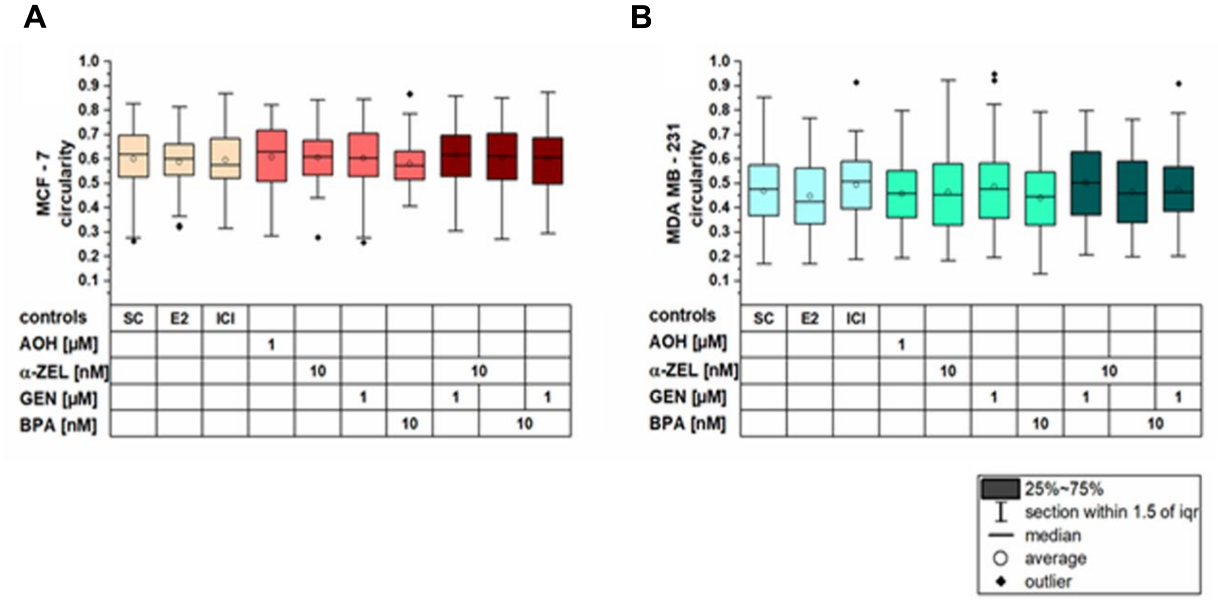

Supplementary Figure 3. Establishment of cellular morphology parameters after 8 h of incubation. Impact of endocrine active substances on circularity of MCF-7 (A), and MDA MB-231 (B). SC: solvent controls, 1 nM E2, 1µM ICI, single substance treatments (1 µM AOH, 10 nM α-ZEL, 1 µM GEN and 10 nM BPA) and mixtures (10 nM α-ZEL + 1µM GEN; 10 nM α-ZEL + 10 nM BPA; 1 µM GEN + 10 nM BPA). Empty circles indicate average values and black diamonds data outliers. Data were obtained from 3 independent cell preparations, quantifying n ≥ 60 cells for each experimental condition.

MCF-7 appearance of cathepsin D

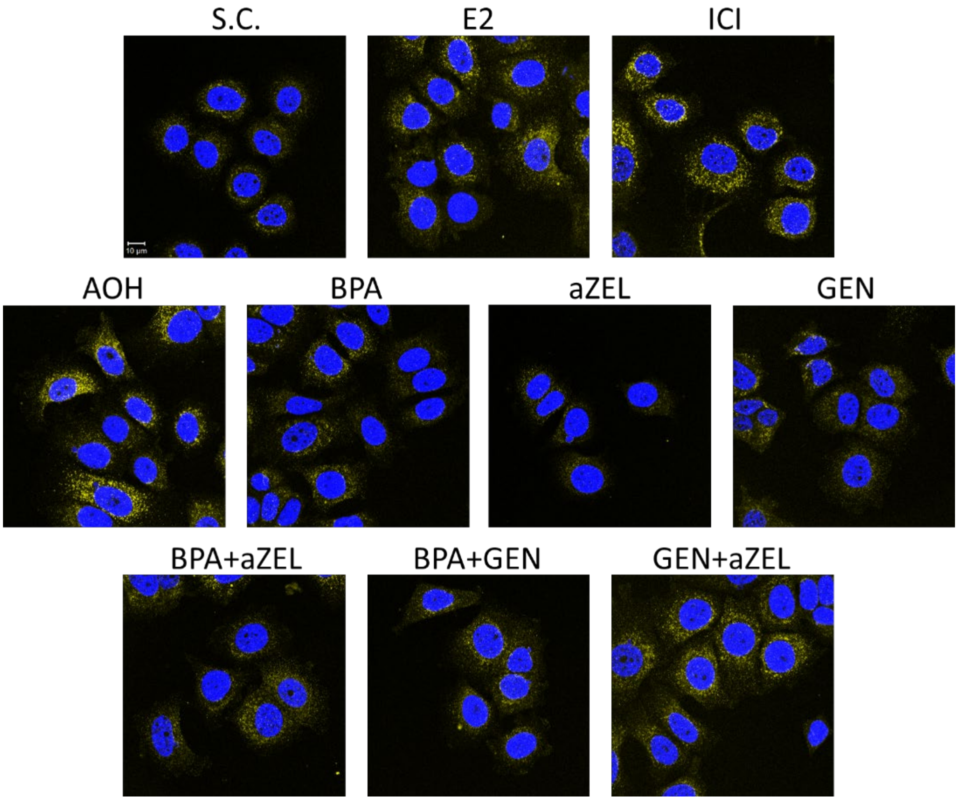

MDA MB-231 appearance of cathepsin D

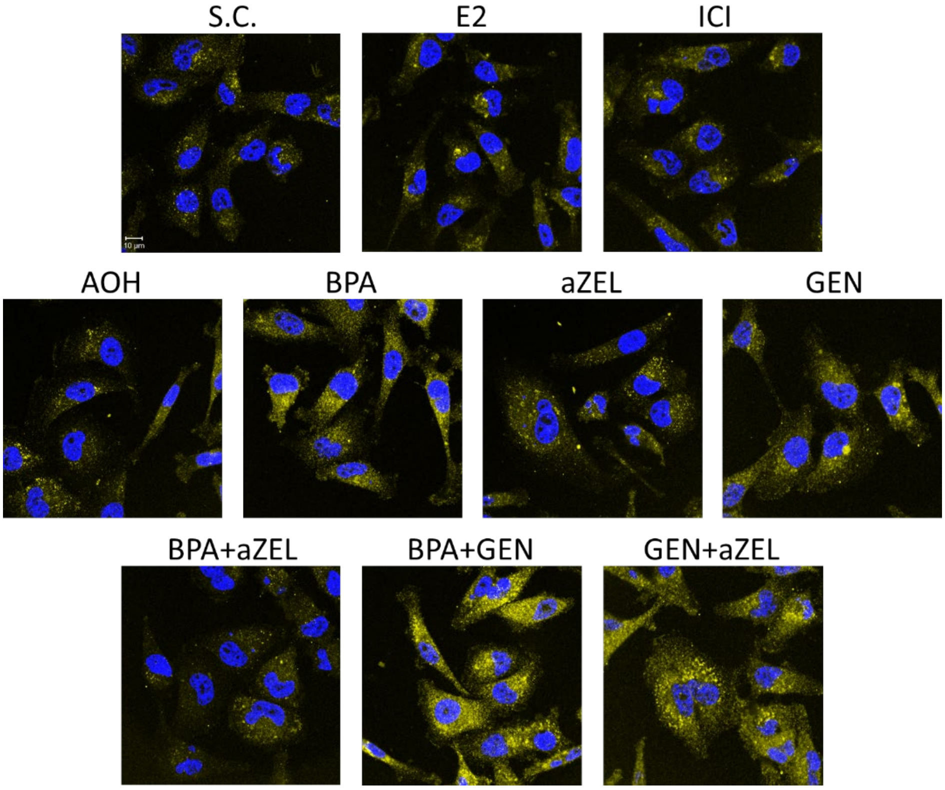

Supplementary Figure 4. Representative appearance of MCF-7 and MDA MB-321 after cathepsin D staining (depicted in yellow). Image panels include solvent controls (SC), E2 (1

nM), ICI (1 µM), AOH (1 µM), α-ZEL (10 nM), GEN (1 µM), BPA (10 nM), and the binary mixtures α-ZEL + GEN, α-ZEL + BPA, and GEN + BPA. Scale bars stand for 10µm.

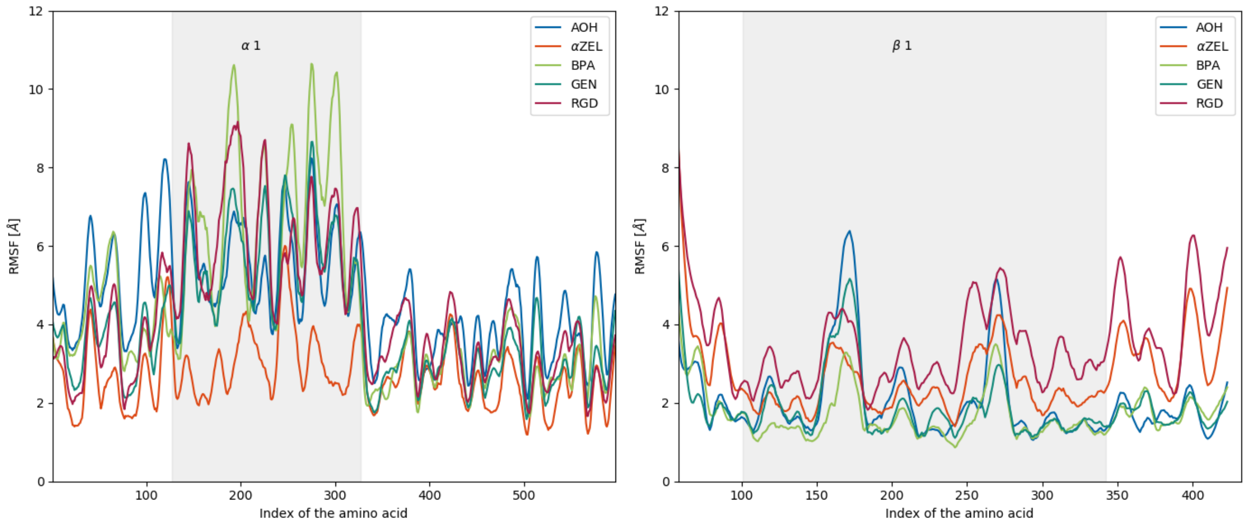

Supplementary Figure 5. RMSF plots of the interaction between the xenoestrogens (AOH, α-ZEL, BPA, GEN) and RDG with integrin α1 and integrin β1.

	AOH	α–ZEL	BPA	GEN	RGD	Water	K^+	Cl^-
Integrin	**10**	0	0	0	0	81384	244	229
Integrin	0	**10**	0	0	0	81335	244	229
Integrin	0	0	**10**	0	0	81381	244	229
Integrin	0	0	0	**10**	0	81361	244	229
Integrin	0	0	0	0	**10**	75849	228	213
Integrin	0	**10**	**10**	0	0	81173	243	228
Integrin	0	**10**	0	**10**	0	81122	243	228
Integrin	0	0	**10**	**10**	0	81171	243	228
Integrin	**10**	0	0	0	**10**	75665	228	213
Integrin	0	**10**	0	0	**10**	75647	227	212
Integrin	0	0	**10**	0	**10**	75615	228	212
Integrin	0	0	0	**10**	**10**	75654	228	213

Supplementary Table 1. Composition of the molecular dynamics simulation systems
